# Diagnostic Accuracy of Serum Antibody, Urine Antibody, and Stool Antigen Tests for *Helicobacter pylori* in Japan: A Systematic Review and Meta‐Analysis

**DOI:** 10.1111/hel.70157

**Published:** 2026-08-16

**Authors:** Chikamasa Ichita, Fumiaki Ishibashi, Yasuhiro Hagiwara, Ayaka Takasu, Yuichiro Kemmoto, Ran Li, Chika Kusano, Tianyi Wei, Yutaka Matsuyama, Nobutake Yamamichi, Manami Inoue, Hideki Ishikawa, Takuji Gotoda

**Affiliations:** ^1^ Department of Gastroenterology Kitasato University School of Medicine Sagamihara Japan; ^2^ Department of Health Data Science, Graduate School of Data Science Yokohama City University Yokohama Japan; ^3^ Digestive Disease Center Showa Medical University Northern Yokohama Hospital Yokohama Japan; ^4^ Department of Biostatistics, School of Public Health, Graduate School of Medicine The University of Tokyo Bunkyō Japan; ^5^ Department of Gastroenterology Cancer Institute Hospital Bunkyō Japan; ^6^ Center for Epidemiology and Preventive Medicine The University of Tokyo Hospital Bunkyō Japan; ^7^ Graduate School of Public Health St. Luke's International University Chūō Japan; ^8^ Department of Molecular‐Targeting Prevention Kyoto Prefectural University of Medicine Kyoto Japan

**Keywords:** diagnostic test accuracy, *Helicobacter pylori*, Japan, meta‐analysis, systematic review

## Abstract

**Background:**

Non‐invasive *Helicobacter pylori* ( *H. pylori* ) tests are widely used in clinical practice in Japan; however, their diagnostic accuracy may vary with the composition of the target population, and no systematic review has synthesized their performance specifically in Japanese populations. Therefore, we conducted a systematic review and meta‐analysis to evaluate the diagnostic accuracy of serum antibody, urine antibody, and stool antigen tests for *H. pylori* infection in Japanese populations.

**Materials and Methods:**

MEDLINE, Embase, and Ichushi‐Web were searched for studies published between 2005 and 2024 evaluating serum antibody, urine antibody, or stool antigen tests in Japanese participants. The primary objective involved evaluation of diagnostic accuracy for current *H. pylori* infection, and the secondary objective involved assessment of accuracy for ever‐infected individuals. Summary estimates were calculated using bivariate random‐effects models. A subgroup analysis was performed under clinically relevant conditions, restricted to studies without previously infected individuals, with composite reference standards, and without application of post hoc cutoff values.

**Results:**

Among 806 publications, 43 studies were selected (serum antibody, 29; urine antibody, six; stool antigen, eight). For diagnosing current *H. pylori* infection, the summary sensitivity/specificity measured 0.922/0.956 for serum antibody, 0.885/0.970 for urine antibody, and 0.928/0.972 for stool antigen tests. For diagnosing ever‐infected individuals, the summary sensitivity/specificity measured 0.908/0.960 for serum antibody and 0.888/0.969 for stool antigen. Under clinically relevant conditions, serum antibody and stool antigen yielded sensitivities/specificities of 0.908/0.968 and 0.935/0.985, respectively. In serum antibody studies, studies with previously infected individuals showed lower accuracy (sensitivity/specificity, 0.876/0.861) than studies without previously infected individuals (0.929/0.967).

**Conclusions:**

Serum antibody and stool antigen tests demonstrated high diagnostic accuracy for current *H. pylori* infection in Japanese populations, although accuracy declined in studies with previously infected individuals.

## 1 Introduction

*Helicobacter pylori* ( *H. pylori* ) infection is a major cause of gastric cancer, peptic ulcer disease, and other upper gastrointestinal conditions [1]. Therefore, accurate diagnosis is fundamental to clinical management, including decisions regarding eradication therapy [2, 3]. Non‐invasive diagnostic methods—including the urea breath test, stool antigen test, and serum/urine antibody tests—are widely used in Japan. The 2024 revised Japanese guidelines recommend selecting among multiple diagnostic tests according to the clinical context and emphasize that test results should be interpreted together with endoscopic findings and clinical information [4].

The diagnostic accuracy of these tests (sensitivity, specificity, and likelihood ratios) is not determined solely by the characteristics of the test kit and may vary according to the composition of the target population, including age, symptom status, and the presence of previously eradicated individuals [5, 6]. Therefore, establishing the diagnostic accuracy of these methods specifically in Japanese populations is essential for their appropriate use in clinical practice. Although several systematic reviews and diagnostic test accuracy meta‐analyses of non‐invasive tests have been reported [7, 8, 9, 10, 11, 12, 13, 14, 15], most reports have involved pooled estimates from multinational data, and the applicability of their findings to clinical practice in Japan may be limited.

We therefore conducted a systematic review and diagnostic test accuracy meta‐analysis restricted to studies of Japanese populations to synthesize the diagnostic accuracy of serum antibody, urine antibody, and stool antigen tests. The primary objective was to evaluate diagnostic accuracy for current *H. pylori* infection, and the secondary objective was to assess diagnostic accuracy for ever‐infected individuals, given the increasing proportion of post‐eradication patients in Japanese clinical populations [16]. To enhance retrieval of domestic evidence, Ichushi‐Web—a database specialized for Japanese medical research—was searched in addition to MEDLINE and Embase. We also examined heterogeneity by the presence of previously infected individuals in the study population, post hoc cutoff use, test kit type, age group, and reference standard composition, and estimated diagnostic accuracy under clinically relevant conditions to inform test selection and result interpretation in Japanese clinical practice.

## 2 Materials and Methods

### 2.1 Protocol and Registration

This systematic review and diagnostic test accuracy meta‐analysis was reported in accordance with the Preferred Reporting Items for Systematic Reviews and Meta‐analyses of Diagnostic Test Accuracy Studies (PRISMA‐DTA) statement [17]. The protocol was prospectively registered on PROSPERO (CRD42025635079). The initial registration (Version 1.0) was published on January 17, 2025, and a subsequent amendment permitting the inclusion of Japanese‐language literature was published as Version 2.0 on May 26, 2025. This amendment was based on the observation that many relevant studies involving Japanese patients were unavailable in English and would have been missed under the original registration criteria.

### 2.2 Eligibility Criteria

Eligibility criteria were defined according to the framework of participants, index test, reference standard, and study design. Participants comprised individuals who underwent *H. pylori* infection testing for clinical or research purposes in Japan, regardless of underlying disease status. Studies evaluating tests for eradication confirmation (assessment of therapeutic efficacy following eradication therapy) were excluded. The index tests were serum antibody tests, urine antibody tests, and stool antigen tests. These represent non‐invasive and easily administered tests with potential applicability to mass screening. The urea breath test was excluded as an index test because its diagnostic accuracy is extremely high, and frequent use occurs as a reference standard. Studies employing endoscopic artificial intelligence diagnosis, genetic/molecular‐based tests, or antibody tests not widely used in clinical practice were also excluded. The type of reference standard was not restricted a priori; however, studies in which the same test method as the index test served as the reference standard (e.g., a study evaluating the accuracy of one serum antibody test using another serum antibody test as the reference) were excluded. With regard to study design, cross‐sectional studies, cohort studies, case–control studies, and randomized controlled trials that reported diagnostic accuracy data (true positive, false positive, false negative, and true negative values, or sensitivity and specificity calculable from these values) for *H. pylori* infection were eligible. Reviews, conference abstracts without sufficient data, and non‐original reports were excluded.

### 2.3 Information Sources and Search Strategy

Three databases—MEDLINE, Embase, and Ichushi‐Web—were systematically searched for literature published between January 1, 2005, and December 31, 2024. Ichushi‐Web is a database specializing in Japanese medical research and was included to ensure comprehensive retrieval of Japanese‐language publications not captured by English‐language databases alone. The search strategy combined terms related to *H. pylori* infection, diagnostic test methods, and diagnostic accuracy, with a geographic filter for Japan applied to the MEDLINE search. The detailed search strategies for each database are presented in Table S1.

### 2.4 Study Selection

Study selection was conducted in two stages. In the first stage, two reviewers (YK and AT) independently screened titles and abstracts. In the second stage, two different reviewers (CI and FI) independently evaluated the full text of potentially eligible studies and determined eligibility. At both stages, disagreements were resolved by a senior researcher (CK).

### 2.5 Data Extraction

The following information was extracted from each included study: first author, publication year, study design, characteristics of the study population (age, symptoms, screening/clinical setting), sample size, type, product name, and cutoff value of the index test, type of reference standard, presence and handling of previously infected individuals, and 2 × 2 contingency table data (true positive, false positive, false negative, and true negative). Data extraction was performed by CI and independently verified by FI.

### 2.6 Risk of Bias Assessment

Risk of bias and applicability concerns for all included studies were assessed using the Quality Assessment of Diagnostic Accuracy Studies 2 (QUADAS‐2) tool [18]. Assessments were conducted independently by CI and FI, with disagreements resolved by CK.

### 2.7 Diagnostic Objectives, Subgroup Analyses, and Clinically Relevant Conditions

For each index test, pooled estimates were calculated according to two diagnostic objectives. The first was the discriminative ability for “current *H. pylori* infection” versus “uninfected plus previously infected” (identification of currently infected individuals). The second involved the discriminative ability for “currently infected plus previously infected” versus “ *H. pylori* ‐naïve” (identification of ever‐infected individuals). Because multiple index test kits were often evaluated simultaneously in the same study population, a single study could generate multiple data points. When results for multiple cutoff values were reported within the same study, the result based on the manufacturer‐recommended cutoff value was adopted for the primary analysis. Subgroup analyses were performed for the following factors considered potential sources of heterogeneity in diagnostic accuracy: (1) presence of previously infected individuals in the study population, (2) use of post hoc cutoff values, (3) test kit type, categorized as enzyme immunoassay (EIA; E‐plate or non‐E‐plate) or particle agglutination/latex agglutination (PA/LA) for serum antibody tests, and as EIA or immunochromatographic assay (ICA) for urine antibody and stool antigen tests, (4) age group of the target population (children vs. adults), and (5) reference standard composition (composite vs. single). Studies were categorized according to the presence or absence of previously infected individuals in the study population. For serum antibody tests, additional subgroup analyses by test kit type were performed after stratification according to the presence or absence of previously infected individuals. For stool antigen tests, additional analyses were performed for EIA‐based assays by separating bioluminescent enzyme immunoassay (BLEIA) from other EIA tests because BLEIA is analytically distinct from conventional EIA. The influence of a polyclonal antibody‐based stool antigen EIA was also assessed by excluding this assay from the EIA subgroup analysis because this older assay type may have different diagnostic performance, particularly specificity, from currently used monoclonal antibody‐based stool antigen tests. Furthermore, an analysis under clinically relevant conditions was performed. In routine clinical practice, individuals with a history of eradication can be excluded through medical interview, composite reference standards combining multiple tests are standard, and post hoc cutoff values determined within individual studies are not used. Therefore, the analysis was restricted to studies without previously infected individuals, with composite reference standards combining multiple tests, and without facility‐specific post hoc cutoff values. This analysis was intended to estimate diagnostic accuracy under these conditions.

### 2.8 Statistical Analysis

The meta‐analysis was performed using a bivariate random‐effects model for sensitivity and specificity. As the primary meta‐analysis model, a generalized linear (logistic) mixed‐effects model incorporating study‐specific, normally distributed random effects with an unstructured variance–covariance matrix was used. However, this model occasionally failed to converge due to sparse data or a limited number of studies. In such cases, alternative models were applied in the following order until model convergence was attained: a linear mixed‐effects model for logit‐transformed sensitivity and specificity, incorporating random effects with an unstructured variance–covariance matrix; a generalized linear (logistic) mixed‐effects model with random effects assuming equal variance for sensitivity and specificity; and a linear mixed‐effects model with random effects assuming equal variance for logit‐transformed sensitivity and specificity. When linear mixed effects models were employed, the Haldane‐Anscombe continuity correction (adding 0.5 to all four cells) was applied to studies with a zero cell. In cases where summary sensitivity and specificity remained unobtainable even after this sequential model‐fitting process, a generalized linear (logistic) fixed‐effect model was ultimately adopted. To account for studies reporting multiple pairs of sensitivity and specificity for a single index test, standard errors were calculated using robust sandwich estimators to adjust for within‐study correlation, regardless of the model type.

Based on the summary sensitivity and specificity obtained from the above process, the positive likelihood ratio (LR+), the negative likelihood ratio (LR−), and the diagnostic odds ratio (DOR) were calculated for each analysis. Confidence intervals (CIs) for these measures were derived using standard errors calculated via the delta method. The summary receiver operating characteristic (SROC) curve was also drawn based on the estimated parameters of bivariate random‐effects models (i.e., summary sensitivity, summary specificity, and the variance–covariance matrix of the random effects) [19]. If the fixed‐effect model was adopted, the SROC curve was not reported.

Bivariate random‐effects and fixed‐effect models were fitted using the GLIMMIX procedure in SAS version 9.4 (SAS Institute Inc., Cary, NC, USA), and all other statistical analyses were conducted using R version 4.5.2 (R Foundation for Statistical Computing, Vienna, Austria).

## 3 Results

### 3.1 Study Selection

**FIGURE 1:**
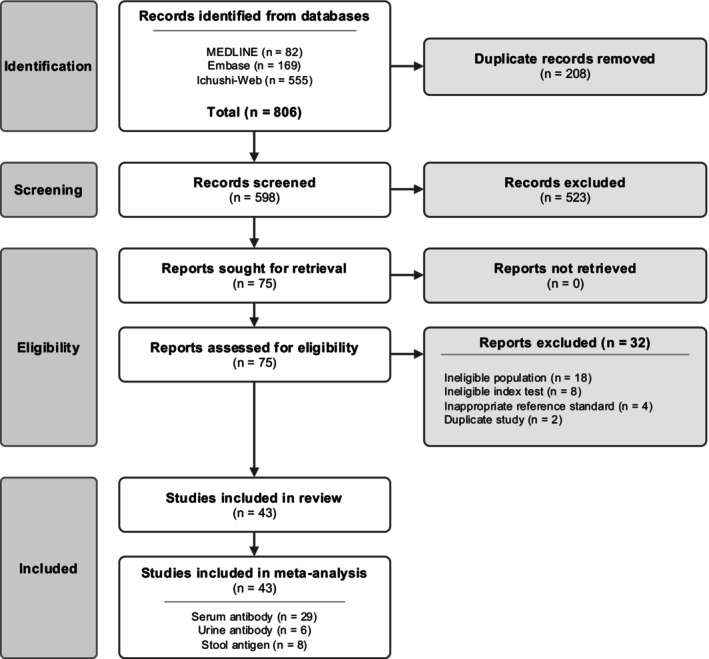
PRISMA flow diagram.

The study selection process is presented in Figure 1. Searches of MEDLINE (PubMed), Embase, and Ichushi‐Web databases identified 806 records published between 2005 and 2024, comprising 82 from MEDLINE, 169 from Embase, and 555 from Ichushi‐Web. After removing duplicates, 598 records were screened by title and abstract. Of these, 523 were excluded, and 75 articles were assessed by full‐text review. A further 32 articles were excluded on the basis of patient‐related criteria (*n* = 18), test method (*n* = 8), reference standard (*n* = 4), and duplicate publication (*n* = 2). Ultimately, 43 studies were included: 29 for serum antibody tests [20, 21, 22, 23, 24, 25, 26, 27, 28, 29, 30, 31, 32, 33, 34, 35, 36, 37, 38, 39, 40, 41, 42, 43, 44, 45, 46, 47, 48], six for urine antibody tests [49, 50, 51, 52, 53, 54], and eight for stool antigen tests [55, 56, 57, 58, 59, 60, 61, 62].

### 3.2 Characteristics of Included Studies

The characteristics of the 43 included studies are presented in Table 1, and the corresponding 2 × 2 contingency data are provided in Table S2. The total number of participants was 11,664 for serum antibody, 1640 for urine antibody, and 1544 for stool antigen tests.

**TABLE 1:** Characteristics of included studies.

No.	Index test category	Author	Year	Population	*N*	Index test (detail)	Reference standard	Diagnostic objective	Past infection info
*Serum antibody*									
1	Serum antibody	Inoue Kazuhiko	2023	Adults	207	L‐type Wako *H. pylori* antibody J (CLEIA), Latex Seiken *H. pylori* antibody (Denka LIA), LZ test Eiken *H. pylori* antibody (LIA), E‐plate Eiken *H. pylori* antibody II (ELISA)	UBT and upper GI endoscopy	Current vs. Others, Ever‐infected vs. Others	No
2	Serum antibody	Takayama Takako	2022	Adults	482	LZ test (LIA)	SAT and endoscopic findings	Current vs. Others, Ever‐infected vs. Others	Yes
3	Serum antibody	Kusano Chika	2021	Children	410	E‐plate Eiken *H. pylori* antibody (ELISA)	SAT	Current vs. Others, Ever‐infected vs. Others	No
4	Serum antibody	Ito Masanori	2020	Adults	2622	LZ test (LIA), E‐plate Eiken *H. pylori* antibody II (EIA), L‐type Wako *H. pylori* antibody J (CLEIA), *H. pylori* Latex Seiken (Denka LIA)	UBT, RUT, SAT, and endoscopic atrophy	Ever‐infected vs. Others	Yes
5	Serum antibody	Ueno Yusuke	2020	Adults	200	LZ test Eiken *H. pylori* antibody II (serum IgG, LIA)	Upper GI endoscopy and UBT	Current vs. Others, Ever‐infected vs. Others	Yes, No
6	Serum antibody	Kodama Masaaki	2019	Adults	152	Denka *H. pylori* Latex Seiken (LIA), Kit A: E‐plate II Eiken (ELISA), Kit B: Premier *H. pylori* kit (ELISA)	Endoscopic gastric atrophy, RUT, and culture	Current vs. Others, Ever‐infected vs. Others	No
7	Serum antibody	Kawai Sayo	2019	Adults	200	E‐plate Eiken *H. pylori* antibody II (ELISA), *H. pylori* IgG Seiken (EIA), IZ test Eiken *H. pylori* Antibody (IZ), *H. pylori* Latex Seiken (LIA)	UBT	Current vs. Others, Ever‐infected vs. Others	No
8	Serum antibody	Tsutsumi Koshiro	2018	Children	209	LZ test (LIA)	SAT	Current vs. Others, Ever‐infected vs. Others	No
9	Serum antibody	Miura Mika	2018	Adults	698	LZ test Eiken *H. pylori* antibody (LIA)	UBT and endoscopic gastric atrophy	Current vs. Others	Yes
10	Serum antibody	Kawai Takashi	2018	Adults	2591	E‐plate serum *H. pylori* antibody (ELISA)	UBT, HpSA, RUT, and upper GI endoscopy	Ever‐infected vs. Others	Yes
11	Serum antibody	Kobayashi Chiaki	2017	Adults	62	SphereLight *H. pylori* antibody J (CLEIA), E‐plate Eiken *H. pylori* antibody (ELISA)	Composite: serum antibody (two methods), SAT, histology, and UBT	Current vs. Others, Ever‐infected vs. Others	No
12	Serum antibody	Kaihara Kazumi	2017	Adults	116	LZ test (LIA), E‐plate Eiken *H. pylori* antibody II (ELISA)	UBT and upper GI endoscopy	Current vs. Others, Ever‐infected vs. Others	No
13	Serum antibody	Inui Masayuki	2017	Adults	256	Wako LIA (CLEIA), Denka LIA, Eiken LZ (LIA)	Composite: endoscopy, RUT, UBT, and SAT	Current vs. Others, Ever‐infected vs. Others	Yes
14	Serum antibody	Aoyama Nobuo	2017	Adults	261	E‐plate (ELISA), LZ test (LIA)	RUT, UBT, endoscopy, and histopathology	Current vs. Others, Ever‐infected vs. Others	No
15	Serum antibody	Furuta Takahisa	2016	Adults	228	Novel latex turbidimetric IgG (prototype RD‐11‐04), Conventional latex (LIA), Conventional EIA	Endoscopic gastric atrophy (Kimura‐Takemoto) and RUT	Current vs. Others, Ever‐infected vs. Others	No
16	Serum antibody	Osumi Hiroki	2016	Adults	205	LZ serum antibody (LIA), E‐plate Eiken (ELISA)	UBT	Current vs. Others, Ever‐infected vs. Others	No
17	Serum antibody	Kodama Masaaki	2016	Adults	101	Novel Denka latex kit (LIA), LZ test (LIA), E‐plate (ELISA)	Endoscopic atrophy, RUT, and culture	Current vs. Others, Ever‐infected vs. Others	No
18	Serum antibody	Shiwa Masaaki	2016	Adults	100	SphereLight *H. pylori* antibody J (CLEIA), E‐plate Eiken *H. pylori* antibody II (ELISA)	Histological examination	Current vs. Others, Ever‐infected vs. Others	No
19	Serum antibody	Inui Masayuki	2016	Adults	273	SphereLight *H. pylori* IgG J (CLEIA)	Composite: RUT, UBT, monoclonal SAT, or histology, combined with endoscopy	Current vs. Others, Ever‐infected vs. Others	Yes
20	Serum antibody	Inui Masayuki	2016	Adults	142	Wako LIA‐HP serum IgG antibody (CLEIA)	Composite: RUT, UBT, SAT, urine antibody, and endoscopy	Current vs. Others, Ever‐infected vs. Others	No
21	Serum antibody	Inui Masayuki	2016	Adults	232	L‐type Wako *H. pylori* antibody J (CLEIA), SphereLight *H. pylori* antibody J (CLEIA), LZ test Eiken *H. pylori* antibody (LIA), E‐plate Eiken *H. pylori* antibody II (ELISA)	Upper GI endoscopy, RUT, UBT, and SAT	Current vs. Others, Ever‐infected vs. Others	Yes
22	Serum antibody	Inoue Kazuhiko	2015	Adults	255	LZ test (LIA)	E‐plate and UBT	Current vs. Others, Ever‐infected vs. Others	No
23	Serum antibody	Kodama Masaaki	2015	Adults	102	Denka latex (LIA), Conventional EIA	Endoscopic atrophy, RUT, and culture	Current vs. Others, Ever‐infected vs. Others	No
24	Serum antibody	Inui Masayuki	2015	Adults	92	E‐plate, LZ, SphereLight J (CLEIA)	Composite: RUT, UBT, urine antibody, SAT, endoscopy, and eradication history	Current vs. Others, Ever‐infected vs. Others	Yes
25	Serum antibody	Furuta Takahisa	2015	Adults	190	Denka EIA IgG antibody kit (prototype), Conventional serum anti‐ *H. pylori* IgG EIA kit	Endoscopic gastric atrophy and RUT	Current vs. Others, Ever‐infected vs. Others	No
26	Serum antibody	Ueda Junko	2014	Children	73	E‐plate Eiken serum IgG antibody (ELISA)	Stool antigen test (HpSA ELISA)	Current vs. Others, Ever‐infected vs. Others	No
27	Serum antibody	Iino Chikara	2013	Adults	994	E‐plate IgG antibody (ELISA)	SAT (monoclonal ELISA; Testmate)	Current vs. Others, Ever‐infected vs. Others	No
28	Serum antibody	Matsuhisa Takeshi	2006	Adults	80	E‐plate Eiken *H. pylori* antibody (ELISA), Determiner *H. pylori* antibody/HM‐CAP (ELISA), Determiner *H. pylori* antibody J/JHM‐CAP (ELISA)	Histopathology (H&E, Giemsa, immunohistochemistry)	Current vs. Others, Ever‐infected vs. Others	No
29	Serum antibody	Okuda Masumi	2005	Children	131	HM‐CAP (EIA)	HpSA and UBT	Current vs. Others, Ever‐infected vs. Others	No
*Urine antibody*									
30	Urine antibody	Mabe Katushiro	2017	Children	780	URINELISA (Otsuka; ELISA)	UBT, SAT, and serum antibody	Current vs. Others, Ever‐infected vs. Others	No
31	Urine antibody	Inoue Kazuhiko	2017	Adults	116	RAPIRUN *H. pylori* antibody stick (ICA), URINELISA *H. pylori* antibody (ELISA)	UBT	Current vs. Others, Ever‐infected vs. Others	No
32	Urine antibody	Okuda Masami	2017	Children	184	Rapid urine *H. pylori* antibody (RAPIRUN; ICA), Urine *H. pylori* ELISA (u‐HpELISA)	Serum antibody ELISA	Current vs. Others, Ever‐infected vs. Others	No
33	Urine antibody	Shimoyama Tadashi	2017	Adults	359	RAPIRUN (Otsuka; ICA), URINELISA (Otsuka; ELISA)	UBT and monoclonal SAT	Current vs. Others, Ever‐infected vs. Others	No
34	Urine antibody	Okuda Masumi	2013	Children	101	URINELISA (Otsuka; ELISA), RAPIRUN (Otsuka; ICA)	UBT and HpSA	Current vs. Others, Ever‐infected vs. Others	No
35	Urine antibody	Okuda Masumi	2004	Children	100	Urine *H. pylori* ELISA (URINELISA)	UBT and HpSA	Current vs. Others, Ever‐infected vs. Others	No
*Stool antigen*									
36	Stool antigen	Kubo Kimitoshi	2024	Adults	1055	Eiken *H. pylori* antigen kit (EIA)	Endoscopy, eradication history, serum antibody, and serum PG II	Current vs. Others, Ever‐infected vs. Others	Yes
37	Stool antigen	Furuta Takahisa	2022	Adults	120	QuickNavi *H. pylori* (ICA)	Composite: culture, PCR, UBT, RUT, and serum pepsinogen	Current vs. Others, Ever‐infected vs. Others	No
38	Stool antigen	Shin Ito	2020	Adults	133	Eiken *H. pylori* antigen BLEIA, Stool antigen test (EIA; Kit A)	Composite: endoscopy, UBT, and serum IgG antibody	Current vs. Others, Ever‐infected vs. Others	No
39	Stool antigen	Okuda M	2014	Children	52	Testmate Pylori Antigen/TPAg (monoclonal EIA)	Quantitative real‐time PCR	Current vs. Others, Ever‐infected vs. Others	No
40	Stool antigen	Naomi Sakaedani	2009	Adults and children	62	Premier Platinum HpSA plus (ELISA)	SAT (EIA; Testmate) and UBT	Current vs. Others, Ever‐infected vs. Others	No
41	Stool antigen	Kayoko Asahi	2007	Adults	30	Testmate Pylori Antigen EIA (catalase‐specific monoclonal)	UBT	Current vs. Others, Ever‐infected vs. Others	No
42	Stool antigen	Sakamoto Teruhiko	2007	Adults and children	49	Monoclonal *H. pylori* stool antigen test (mHpSAT)	Composite: monoclonal SAT, RUT, serum antibody, and histology	Current vs. Others, Ever‐infected vs. Others	No
43	Stool antigen	Koike Michio	2005	Children	43	Meridian HpSA ELISA, Testmate Pylori Antigen EIA	UBT	Current vs. Others, Ever‐infected vs. Others	No

Abbreviations: CLEIA, chemiluminescent enzyme immunoassay; EIA, enzyme immunoassay; ELISA, enzyme‐linked immunosorbent assay; GI, gastrointestinal; H&E, hematoxylin and eosin; HpSA, *Helicobacter pylori* stool antigen; ICA, immunochromatographic assay; LIA, latex immunoassay; PCR, polymerase chain reaction; PG, pepsinogen; RUT, rapid urease test; SAT, stool antigen test; UBT, urea breath test.

### 3.3 Risk of Bias Assessment (QUADAS‐2)

Risk of bias and applicability were assessed using QUADAS‐2 for all 43 included studies (Table S3). In the risk of bias assessment, 11 studies were judged to be high risk and six as unclear in the patient selection domain, representing the most problematic domain among the four. The main reasons included non‐consecutive patient enrollment and case–control study designs. For the index test domain, 42 studies were rated as low risk, and all 43 studies were rated as low risk for the flow and timing domain. For the reference standard domain, seven studies were rated as high risk and one as unclear, mainly because a single reference standard was used. In the applicability assessment, 25 studies were judged to have concerns regarding patient selection because this review considered studies with previously infected individuals to be less applicable to the target population. Five studies had concerns regarding the index test, and one was unclear, corresponding to studies that used research‐grade test methods. Twenty‐one studies were judged to have concerns regarding the reference standard, most of which used a single reference standard. These concerns were further explored in subgroup analyses.

### 3.4 Main Analysis Results

#### 3.4.1 Serum Antibody Tests (29 Studies)

**FIGURE 2:**
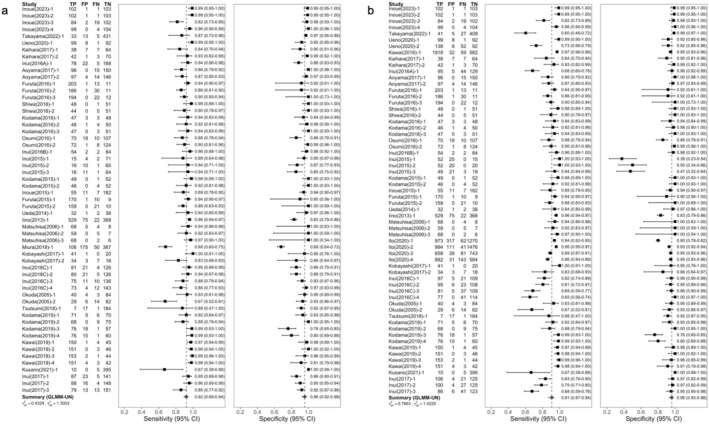
Forest plots of serum antibody tests. (a) Current infection versus others; (b) ever‐infected versus others. *τ*_sn_^2^ and *τ*_sp_^2^ represent variances of random effects for sensitivity and specificity. CI, confidence interval; FN, false negative; FP, false positive; GLMM‐UN, generalized linear mixed‐effects model incorporating random effects with an unstructured variance–covariance matrix; TN, true negative; TP, true positive.

The diagnostic accuracy results for serum antibody tests are presented in Table 2 and Figure 2 (Summary ROC curves are provided in Figure S1). For detecting current infection, the summary sensitivity was 0.922 (95% CI: 0.895–0.942) and the specificity was 0.956 (95% CI: 0.921–0.976). The LR+ was 20.739 (95% CI: 11.478–37.471), the LR− was 0.082 (95% CI: 0.061–0.111), and the DOR was 253.003 (95% CI: 126.159–507.378). For identifying ever‐infected individuals, the summary sensitivity was 0.908 (95% CI: 0.869–0.936) and the specificity was 0.960 (95% CI: 0.932–0.977). The LR+ was 22.927 (95% CI: 13.326–39.444), the LR− was 0.096 (95% CI: 0.068–0.137), and the DOR was 238.483 (95% CI: 129.103–440.535).

**TABLE 2:** Summary estimates of diagnostic accuracy for each index test.

Index test	Diagnostic objective	No. of studies	No. of data points	Sn (95% CI)	Sp (95% CI)	LR+ (95% CI)	LR− (95% CI)	DOR (95% CI)
Serum	Current vs. Others	25	57	0.922 (0.895–0.942)	0.956 (0.921–0.976)	20.739 (11.478–37.471)	0.082 (0.061–0.111)	253.003 (126.159–507.378)
Serum	Ever‐infected vs. Others	26	62	0.908 (0.869–0.936)	0.960 (0.932–0.977)	22.927 (13.326–39.444)	0.096 (0.068–0.137)	238.483 (129.103–440.535)
Urine	Current vs. Others	6	12	0.885 (0.710–0.960)	0.970 (0.728–0.997)	29.016 (2.755–305.556)	0.119 (0.045–0.319)	243.618 (24.319–2440.490)
Urine	Ever‐infected vs. Others	6	12	0.885 (0.710–0.960)	0.970 (0.728–0.997)	29.016 (2.755–305.556)	0.119 (0.045–0.319)	243.618 (24.319–2440.490)
Stool	Current vs. Others	8	11	0.928 (0.883–0.956)	0.972 (0.937–0.988)	33.044 (14.427–75.685)	0.074 (0.045–0.122)	443.767 (161.998–1215.627)
Stool	Ever‐infected vs. Others	8	11	0.888 (0.659–0.970)	0.969 (0.932–0.986)	29.014 (13.973–60.243)	0.115 (0.033–0.400)	251.640 (78.455–807.125)

Abbreviations: CI, confidence interval; DOR, diagnostic odds ratio; LR−, negative likelihood ratio; LR+, positive likelihood ratio; Sn, sensitivity; Sp, specificity.

#### 3.4.2 Urine Antibody Tests (Six Studies)

**FIGURE 3:**
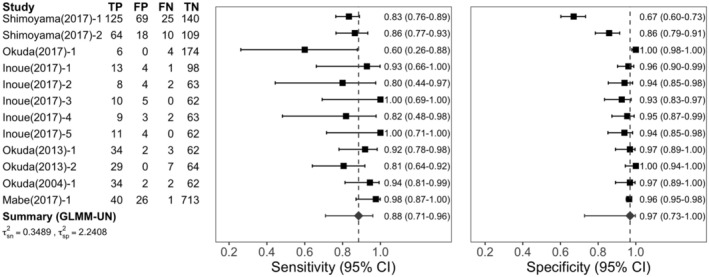
Forest plot of urine antibody tests. Because previously infected individuals were not present, results for current infection versus others and ever‐infected versus others are identical; only current infection versus others is presented. τsn2 and τsp2 represent variances of random effects for sensitivity and specificity. CI, confidence interval; FN, false negative; FP, false positive; GLMM‐UN, generalized linear mixed‐effects model incorporating random effects with an unstructured variance–covariance matrix; TN, true negative; TP, true positive.

The results for urine antibody tests are presented in Table 2 and Figure 3 (Summary ROC curve is provided in Figure S2). Because previously infected individuals were not present in the urine antibody studies, the diagnostic classifications for “current infection vs. others” and “ever‐infected vs. others” were identical; therefore, only the former is presented. For detecting current infection, the summary sensitivity was 0.885 (95% CI: 0.710–0.960) and the specificity was 0.970 (95% CI: 0.728–0.997). The LR+ was 29.016 (95% CI: 2.755–305.556), the LR− was 0.119 (95% CI: 0.045–0.319), and the DOR was 243.618 (95% CI: 24.319–2440.490). The wide CIs reflect substantial heterogeneity.

#### 3.4.3 Stool Antigen Tests (Eight Studies)

**FIGURE 4:**
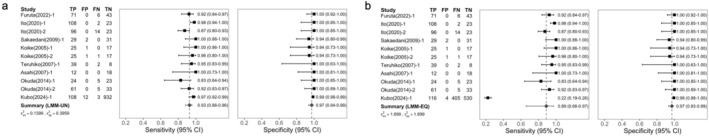
Forest plots of stool antigen tests. (a) Current infection versus others; (b) ever‐infected versus others. *τ*_sn_^2^ and *τ*_sp_^2^ represent variances of random effects for sensitivity and specificity. CI, confidence interval; FN, false negative; FP, false positive; LMM‐EQ, linear mixed‐effects model with random effects assuming equal variance for sensitivity and specificity; LMM‐UN, linear mixed‐effects model incorporating random effects with an unstructured variance–covariance matrix; TN, true negative; TP, true positive.

The results for stool antigen tests are presented in Table 2 and Figure 4 (Summary ROC curves are provided in Figure S3). For detecting current infection, the summary sensitivity was 0.928 (95% CI: 0.883–0.956) and the specificity was 0.972 (95% CI: 0.937–0.988). The LR+ was 33.044 (95% CI: 14.427–75.685), the LR− was 0.074 (95% CI: 0.045–0.122), and the DOR was 443.767 (95% CI: 161.998–1215.627). For identifying ever‐infected individuals, the summary sensitivity was 0.888 (95% CI: 0.659–0.970) and the specificity was 0.969 (95% CI: 0.932–0.986). The LR+ was 29.014 (95% CI: 13.973–60.243), the LR− was 0.115 (95% CI: 0.033–0.400), and the DOR was 251.640 (95% CI: 78.455–807.125).

### 3.5 Subgroup Analyses

#### 3.5.1 Subgroup Analysis by Presence of Previously Infected Individuals

Detailed results are presented in Table S4 and Figures S4 and S5. In studies with previously infected individuals, the sensitivity for current infection was 0.876 (95% CI, 0.712–0.953) and the specificity was 0.861 (95% CI, 0.705–0.942). In studies without previously infected individuals, the sensitivity was 0.929 (95% CI, 0.901–0.950) and the specificity was 0.967 (95% CI, 0.934–0.984). For urine antibody tests, previously infected individuals were not present in any study; therefore, this analysis was not performed. For stool antigen tests, only one study had previously infected individuals, which was insufficient for subgroup comparison.

#### 3.5.2 Subgroup Analysis by Post Hoc Cutoff Use

Detailed results are presented in Table S5 and Figures S6 and S7. In studies without post hoc cutoff use, the sensitivity for current infection was 0.926 (95% CI, 0.902–0.944) and the specificity was 0.961 (95% CI, 0.931–0.978). In studies with post hoc cutoff use, the sensitivity was 0.886 (95% CI, 0.552–0.980) and the specificity was 0.836 (95% CI, 0.601–0.946), although the CIs were wide. For urine antibody and stool antigen tests, no studies used post hoc cutoff values; therefore, this analysis was not performed.

#### 3.5.3 Subgroup Analysis by Test Kit Type

Detailed results are presented in Tables S6a and S6c and Figures S8–S13. Studies using EIA E‐plate yielded a sensitivity of 0.915 (95% CI: 0.884–0.938) and specificity of 0.993 (95% CI: 0.962–0.999) for current infection, demonstrating high diagnostic accuracy. Studies using other EIA kits showed a sensitivity of 0.959 (95% CI: 0.911–0.981) and a specificity of 0.933 (95% CI: 0.749–0.985). Studies using PA/LA showed a sensitivity of 0.923 (95% CI: 0.878–0.952) and a specificity of 0.937 (95% CI: 0.897–0.962). For urine antibody tests, EIA‐based studies yielded a sensitivity of 0.940 (95% CI: 0.880–0.971) and a specificity of 0.947 (95% CI: 0.890–0.975) for current infection, whereas ICA‐based studies showed a sensitivity of 0.794 (95% CI: 0.465–0.945) and a specificity of 0.974 (95% CI: 0.510–0.999), with wide CIs. For stool antigen tests, EIA‐based studies yielded a sensitivity of 0.954 (95% CI: 0.910–0.977) and a specificity of 0.987 (95% CI: 0.971–0.994) for current infection, demonstrating high diagnostic accuracy. Additional subgroup analyses of serum antibody tests by test kit type after stratification by the presence of previously infected individuals are presented in Table S6b. Among studies without previously infected individuals, serum antibody tests showed high diagnostic accuracy across test kit types. Among studies with previously infected individuals, specificity tended to be lower, particularly for non‐E‐plate EIA and PA/LA. Additional analyses of stool antigen EIA tests after separating BLEIA and excluding polyclonal HpSA are presented in Table S6c. EIA tests excluding BLEIA showed high diagnostic accuracy, although sensitivity was lower than that of BLEIA. Because polyclonal HpSA is an older antibody‐based assay, the EIA subgroup was further evaluated after excluding this assay. The point estimates were comparable to those obtained after excluding BLEIA alone.

#### 3.5.4 Subgroup Analysis by Age Group

Detailed results are presented in Table S7 and Figures S14–S19. In adult populations, the sensitivity and specificity for current infection were 0.928 (95% CI, 0.903–0.947) and 0.949 (95% CI, 0.912–0.971), respectively. In pediatric populations, the sensitivity was lower at 0.827 (95% CI, 0.515–0.956), whereas the specificity was 0.980 (95% CI, 0.466–1.000), with wide CIs. For urine antibody tests, adult studies showed imprecise estimates with wide CIs, whereas pediatric studies showed a sensitivity of 0.894 (95% CI, 0.747–0.960) and a specificity of 0.973 (95% CI, 0.943–0.987) for current infection. For stool antigen tests, the diagnostic accuracy was high in both adults and children. In adults, the sensitivity and specificity for current infection were 0.938 (95% CI, 0.900–0.963) and 0.989 (95% CI, 0.979–0.994), respectively, whereas in children the sensitivity and specificity were 0.933 (95% CI, 0.494–0.995) and 0.980 (95% CI, 0.770–0.999), respectively.

#### 3.5.5 Subgroup Analysis by Reference Standard Composition

Detailed results are presented in Table S8 and Figures S20–S25. For current infection, studies using composite reference standards yielded a sensitivity of 0.916 (95% CI, 0.888–0.938) and a specificity of 0.943 (95% CI, 0.886–0.972), whereas studies using single reference standards showed a sensitivity of 0.923 (95% CI, 0.868–0.956) and a specificity of 0.965 (95% CI, 0.899–0.988), with no substantial differences between the two groups. For urine antibody tests, studies using composite reference standards yielded a sensitivity of 0.920 (95% CI, 0.770–0.975) and a specificity of 0.946 (95% CI, 0.761–0.990), whereas studies using single reference standards showed a sensitivity of 0.806 (95% CI, 0.304–0.975) and a specificity of 0.978 (95% CI, 0.781–0.998), although the CIs were wide. For stool antigen tests, the diagnostic accuracy was high regardless of reference standard composition. Studies using composite reference standards showed a sensitivity of 0.935 (95% CI, 0.893–0.961) and a specificity of 0.985 (95% CI, 0.817–0.999), whereas studies using single reference standards showed a sensitivity of 0.952 (95% CI, 0.864–0.984) and a specificity of 0.976 (95% CI, 0.927–0.992). For stool antigen tests, the diagnostic accuracy remained high in both groups.

### 3.6 Analysis Under Clinically Relevant Conditions

An analysis restricted to studies without previously infected individuals, with composite reference standards, and without the use of post hoc cutoff values was conducted. Detailed results are presented in Table S9 and Figures S26–S30. For serum antibody tests, the sensitivity for current infection was 0.908 (95% CI, 0.855–0.943) and the specificity was 0.968 (95% CI, 0.928–0.986). Compared with the main analysis, the specificity showed a modest increase. For urine antibody tests, the sensitivity for current infection was 0.920 (95% CI, 0.770–0.975) and the specificity was 0.946 (95% CI, 0.761–0.990), although the CIs remained wide. For stool antigen tests, the sensitivity for current infection was 0.935 (95% CI, 0.893–0.961) and the specificity was 0.985 (95% CI, 0.817–0.999).

## 4 Discussion

This systematic review and diagnostic test accuracy meta‐analysis synthesized data from 43 studies conducted in Japanese populations and provides quantitative evidence on the diagnostic accuracy of serum antibody, urine antibody, and stool antigen tests. Serum antibody tests (sensitivity/specificity, 0.922/0.956) and stool antigen tests (sensitivity/specificity, 0.928/0.972) both demonstrated high accuracy for identifying current infection, with stool antigen tests showing particularly high specificity. Urine antibody tests also showed generally favorable performance (sensitivity/specificity, 0.885/0.970), although the number of studies was limited and CIs were wide. In the analysis under clinically relevant conditions, restricted to studies without previously infected individuals, with composite reference standards, and without the use of post hoc cutoff values, serum antibody tests (sensitivity/specificity, 0.908/0.968) showed a modest improvement in specificity, and stool antigen tests (sensitivity/specificity, 0.935/0.985) maintained high accuracy. Furthermore, in serum antibody studies, the presence of previously infected individuals in the study population was associated with lower diagnostic accuracy, with sensitivity/specificity of 0.876/0.861 in studies with previously infected individuals compared with 0.929/0.967 in studies without previously infected individuals. This finding suggests that the presence of previously infected individuals represented an important source of heterogeneity in diagnostic accuracy.

Several systematic reviews and meta‐analyses of non‐invasive *H. pylori* tests have been reported [7, 8, 9, 10, 11, 12, 13, 14, 15]. However, most have pooled multinational data, and the direct applicability of their findings to the Japanese clinical setting may be limited. In the Cochrane review by Best et al. *H. pylori* infection, without separately addressing past infection, was restricted to initial testing, and used histopathological examination as the reference standard. Moreover, thresholds were not specified in 14 of 34 serology studies and 14 of 29 stool antigen test studies, and summary sensitivity/specificity at a common threshold was not calculated for stool antigen tests [7]. Accordingly, the main comparisons should be interpreted as indirect comparisons based on sensitivity estimates at a fixed specificity of 0.90 and SROC analysis. Because the diagnostic accuracy can vary according to disease prevalence and the spectrum of the target population, sensitivity and specificity may not be invariant across different population settings. Therefore, for application to Japanese populations, the diagnostic accuracy should be evaluated directly in Japanese participants under explicitly defined clinically relevant conditions, including the handling of past infection, the reference standard, and cutoff use, rather than by simply extrapolating multinational pooled estimates.

These findings have direct implications for *H. pylori* testing in Japan. Among the three modalities, serum antibody tests had the largest evidence base under clinically relevant conditions and may represent the most practical for routine use, whereas stool antigen tests may be preferred when accurate identification of current infection is prioritized. The observed decline in serum antibody accuracy in studies with previously infected individuals underscores the need to assess eradication history when interpreting results; in settings where eradication therapy is widespread, results should be interpreted together with clinical information, including birth cohort and treatment history. As *H. pylori* prevalence continues to decline in Japan [63, 64] and gastric cancer is rare in *H. pylori* –naïve individuals [65], accurate identification of infection status through non‐invasive testing is likely to become increasingly important for future screening strategies, and the present data provide foundational evidence for this purpose.

The present study has several limitations. First, in the risk of bias assessment, 11 studies were rated as high risk and six as unclear in the patient selection domain, whereas seven studies were rated as high risk and one as unclear in the reference standard domain. Although concerns regarding applicability were partly explored in subgroup analyses, studies with potential methodological limitations were still included from the perspective of risk of bias. Second, the number of studies on urine antibody and stool antigen tests was limited, precluding adequate evaluation of the effects of past infection and performance by age group for these tests. Third, although restricting the analysis to Japanese participants enhances the relevance of the findings for implementation in Japan, the generalizability of the results to other countries remains limited. Fourth, for serum antibody tests, values below the cutoff but within the high range (so‐called high‐negative values) are associated with current infection or higher gastric cancer risk, suggesting the potential utility of stepwise diagnostic strategies incorporating additional testing [66, 67]. However, the present study did not perform threshold‐specific analyses focused on high‐negative values or evaluate stepwise diagnostic algorithms for such cases. Finally, because the urea breath test was used as a reference standard in many of the included studies, an independent evaluation as an index test was not conducted. Therefore, the findings of this study should be regarded as foundational evidence for implementing non‐invasive *H. pylori* tests in Japan, rather than as a comprehensive comparison of all non‐invasive tests.

## 5 Conclusion

Among non‐invasive *H. pylori* tests in Japanese populations, serum antibody and stool antigen tests demonstrated high diagnostic accuracy for identifying current infection, and urine antibody tests also showed favorable point estimates, although the evidence remains limited. However, the accuracy of serum antibody tests may decline in real‐world settings with previously infected individuals, and diagnostic performance varies across test kit types. These findings underscore the need to consider test‐specific accuracy differences and prior infection history when implementing *H. pylori* tests in Japanese clinical practice.

## Author Contributions

C.I., F.I., C.K., M.I., H.I., and T.G. designed the study. F.I. and C.I. collected the data. A.T. and Y.K. screened the data. F.I., C.I., R.L., Y.H., T.W., Y.M., C.K., M.I., and T.G. analyzed and interpreted the data. C.I. drafted the manuscript. F.I. critically revised the manuscript. T.G. supervised the study. All the authors have approved the final manuscript.

## Funding

This study was supported by the Labour and Welfare Sciences Research Grants (Research for Promotion of Cancer Control Programmes: 24EA1001).

## Ethics Statement

Ethical approval and informed consent were not required because this study was a systematic review and meta‐analysis of previously published data.

## Conflicts of Interest

F.I. received honoraria for lectures from the Fujifilm Corporation and Olympus Corporation. C.K. received honoraria for lectures from the Fujifilm Corporation and Olympus Corporation. T.G. and F.I. received honoraria for lectures from the Fujifilm Corporation, Fujifilm Medical, and Olympus Corporation. The remaining authors declare no conflicts of interest.

## Supporting information


**Table S1:** Search strategy.
**Table S2:** 2 × 2 contingency data for all included study–test combinations.
**Table S3:** Quality assessment of included studies using QUADAS‐2.
**Table S4:** Subgroup analysis by presence of previously infected individuals.
**Table S5:** Subgroup analysis: post hoc cutoff use.
**Table S6a:** Subgroup analysis: test kit type.
**Table S6b:** Subgroup analysis of serum antibody tests by test kit type after stratification by the presence of previously infected individuals.
**Table S6c:** Additional analysis of stool antigen EIA tests after separating BLEIA and excluding polyclonal HpSA.
**Table S7:** Subgroup analysis: age group.
**Table S8:** Subgroup analysis: reference standard composition.
**Table S9:** Analysis under clinically relevant conditions.
**Figure S1:** Summary ROC curves of serum antibody tests.
**Figure S2:** Summary ROC curve of urine antibody tests.
**Figure S3:** Summary ROC curves of stool antigen tests.
**Figure S4:** Forest plots: serum antibody, studies with previously infected individuals.
**Figure S5:** Forest plots: serum antibody, studies without previously infected individuals.
**Figure S6:** Forest plots: serum antibody, without post hoc cutoff use.
**Figure S7:** Forest plots: serum antibody, with post hoc cutoff use.
**Figure S8:** Forest plots: serum antibody, EIA (E‐plate).
**Figure S9:** Forest plots: serum antibody, EIA (non‐E‐plate).
**Figure S10:** Forest plots: serum antibody, PA/LA.
**Figure S11:** Forest plots: urine antibody, EIA.
**Figure S12:** Forest plots: urine antibody, ICA.
**Figure S13:** Forest plots: stool antigen, EIA.
**Figure S14:** Forest plots: serum antibody, adults.
**Figure S15:** Forest plots: serum antibody, children.
**Figure S16:** Forest plots: urine antibody, adults.
**Figure S17:** Forest plots: urine antibody, children.
**Figure S18:** Forest plots: stool antigen, adults.
**Figure S19:** Forest plots: stool antigen, children.
**Figure S20:** Forest plots: serum antibody, composite reference standards.
**Figure S21:** Forest plots: serum antibody, single reference standard.
**Figure S22:** Forest plots: urine antibody, composite reference standards.
**Figure S23:** Forest plots: urine antibody, single reference standard.
**Figure S24:** Forest plots: stool antigen, composite reference standards.
**Figure S25:** Forest plots: stool antigen, single reference standard.
**Figure S26:** Forest plots: serum antibody, clinically relevant conditions.
**Figure S27:** SROC curve: serum antibody, clinically relevant conditions.
**Figure S28:** Forest plots: urine antibody, clinically relevant conditions.
**Figure S29:** SROC curve: urine antibody, clinically relevant conditions.
**Figure S30:** Forest plots: stool antigen, clinically relevant conditions.

## Data Availability

The data supporting the findings of this study were extracted from previously published articles. Additional extracted data are available from the corresponding author upon reasonable request.

## References

[1] P. Malfertheiner , F. Megraud , T. Rokkas , et al., “Management of *Helicobacter pylori* Infection: The Maastricht VI/Florence Consensus Report,” Gut 71, no. 9 (2022): 1724–1762, 10.1136/gutjnl-2022-327745. 35944925

[2] F. Ishibashi , S. Suzuki , M. Nagai , K. Mochida , and T. Morishita , “Optimizing *Helicobacter pylori* Treatment: An Updated Review of Empirical and Susceptibility Test‐Based Treatments,” Gut Liver 17, no. 5 (2023): 684–697, 10.5009/gnl220429. 36843419 PMC10502504

[3] W. D. Chey , C. W. Howden , S. F. Moss , et al., “ACG Clinical Guideline: Treatment of *Helicobacter pylori* Infection,” American Journal of Gastroenterology 119, no. 9 (2024): 1730–1753, 10.14309/ajg.0000000000002968Ta. 39626064

[4] H. Isomoto , T. Shimoyama , M. Ito , et al., “Guidelines for the Management of *Helicobacter pylori* Infection in Japan: 2024 Revised Edition,” Journal of Gastroenterology 61, no. 7 (2026): 841–862. 10.1007/s00535-026-02379-4. 41902905 PMC13283202

[5] M. M. G. Leeflang , P. M. M. Bossuyt , and L. Irwig , “Diagnostic Test Accuracy May Vary With Prevalence: Implications for Evidence‐Based Diagnosis,” Journal of Clinical Epidemiology 62, no. 1 (2009): 5–12, 10.1016/j.jclinepi.2008.04.007. 18778913

[6] N. D. Vijfschagt , H. Burger , M. Y. Berger , et al., “Variation in Sensitivity and Specificity of Diverse Diagnostic Tests Across Health‐Care Settings: A Meta‐Epidemiological Study,” Journal of Clinical Epidemiology 184 (2025): 111816, 10.1016/j.jclinepi.2025.111816. 40339825

[7] L. M. Best , Y. Takwoingi , S. Siddique , et al., “Non‐Invasive Diagnostic Tests for *Helicobacter pylori* Infection,” Cochrane Database of Systematic Reviews 3, no. 3 (2018): CD012080, 10.1002/14651858.CD012080.pub2. 29543326 PMC6513531

[8] Y. Gong , Q. Li , and Y. Yuan , “Accuracy of Testing for Anti‐ *Helicobacter pylori* IgG in Urine for *H. pylori* Infection Diagnosis: A Systematic Review and Meta‐Analysis,” BMJ Open 7, no. 4 (2017): e013248, 10.1136/bmjopen-2016-013248. PMC5719657 28455424

[9] J. P. Gisbert and V. Abraira , “Accuracy of *Helicobacter pylori* Diagnostic Tests in Patients With Bleeding Peptic Ulcer: A Systematic Review and Meta‐Analysis,” American Journal of Gastroenterology 101, no. 4 (2006): 848–863, 10.1111/j.1572-0241.2006.00528.x. 16494583

[10] X. Zhou , J. Su , G. Xu , and G. Zhang , “Accuracy of Stool Antigen Test for the Diagnosis of *Helicobacter pylori* Infection in Children: A Meta‐Analysis,” Clinics and Research in Hepatology and Gastroenterology 38, no. 5 (2014): 629–638, 10.1016/j.clinre.2014.02.001. 24629927

[11] Y. A. Leal , L. L. Flores , E. M. Fuentes‐Pananá , R. Cedillo‐Rivera , and J. Torres , “13C‐Urea Breath Test for the Diagnosis of *Helicobacter pylori* Infection in Children: A Systematic Review and Meta‐Analysis,” Helicobacter 16, no. 4 (2011): 327–337, 10.1111/j.1523-5378.2011.00863.x. 21762274

[12] Y. A. Leal , L. L. Flores , L. B. García‐Cortés , R. Cedillo‐Rivera , and J. Torres , “Antibody‐Based Detection Tests for the Diagnosis of *Helicobacter pylori* Infection in Children: A Meta‐Analysis,” PLoS One 3, no. 11 (2008): e3751, 10.1371/journal.pone.0003751. 19015732 PMC2582133

[13] M. Omar , R. Abu‐Salah , R. Agbareia , et al., “A Comparative Systematic Review and Meta‐Analysis on the Diagnostic Accuracy of Non‐Invasive Tests for *Helicobacter pylori* Detection in Elderly Patients,” Frontiers in Medicine (Lausanne) 10 (2023): 1323113, 10.3389/fmed.2023.1323113. PMC10748425 38143438

[14] M. Silva Luz , C. Tianeze de Castro , F. F. Bueno Lemos , et al., “Stool Antigen Test for *Helicobacter pylori* Infection in Adults: A Meta‐Analysis of Diagnostic Test Accuracy,” Journal of Clinical Gastroenterology 59, no. 5 (2025): 393–404, 10.1097/MCG.0000000000002102. 39928545

[15] J. P. Gisbert , F. de la Morena , and V. Abraira , “Accuracy of Monoclonal Stool Antigen Test for the Diagnosis of *H. pylori* Infection: A Systematic Review and Meta‐Analysis,” American Journal of Gastroenterology 101, no. 8 (2006): 1921–1930, 10.1111/j.1572-0241.2006.00668.x. 16780557

[16] H. Deguchi , A. Uda , and K. Murakami , “Current Status of *Helicobacter pylori* Diagnosis and Eradication Therapy in Japan Using a Nationwide Database,” Digestion 101, no. 4 (2020): 441–449, 10.1159/000500819. 31216549 PMC7384341

[17] M. D. F. McInnes , D. Moher , B. D. Thombs , et al., “Preferred Reporting Items for a Systematic Review and Meta‐Analysis of Diagnostic Test Accuracy Studies: The PRISMA‐DTA Statement,” Journal of the American Medical Association 319, no. 4 (2018): 388–396, 10.1001/jama.2017.19163. 29362800

[18] P. F. Whiting , A. W. S. Rutjes , M. E. Westwood , et al., “QUADAS‐2: A Revised Tool for the Quality Assessment of Diagnostic Accuracy Studies,” Annals of Internal Medicine 155, no. 8 (2011): 529–536, 10.7326/0003-4819-155-8-201110180-00009. 22007046

[19] R. M. Harbord , J. J. Deeks , M. Egger , P. Whiting , and J. A. C. Sterne , “A Unification of Models for Meta‐Analysis of Diagnostic Accuracy Studies,” Biostatistics 8, no. 2 (2007): 239–251, 10.1093/biostatistics/kxl004. 16698768

[20] K. Inoue , N. Hisamoto , and K. Haruma , “A Prospective Study of the Precision of Four Serum *Helicobacter pylori* Antibody Measurement Methods,” Ningen Dock International 10 (2023): 1–11.

[21] T. Takayama , H. Suzuki , K. Okada , et al., “The Optimal Cut‐Off of the Latex Immunoassay (LZ Test) for *Helicobacter pylori* Infection Based on the Stool Antigen Test and *Helicobacter pylori* ‐Associated Gastritis,” Internal Medicine 61, no. 14 (2022): 2103–2109, 10.2169/internalmedicine.8659-21. 35850984 PMC9381343

[22] C. Kusano , T. Gotoda , H. Ikehara , et al., “The Accuracy of the Serum Antibody Test for *Helicobacter pylori* Infection Among Junior High School Students,” Digestion 102, no. 2 (2021): 155–160, 10.1159/000502900. 31505488

[23] M. Ito , N. Aoyama , T. Furuta , et al., “Evaluation of Gastric Cancer Risk by Optimized Serum Antibody Titers Against *H. pylori* : A Multi‐Center Retrospective Study (Second Report),” Journal of the Japan Society for Helicobacter Research 22 (2020): 51–57.

[24] Y. Ueno , N. Sasaki , and K. Inoue , “Evaluation of “LZ TEST ‘EIKEN’ *H. pylori* Antibody II” for Usefulness,” Japanese Journal of Medical and Pharmaceutical Science 77 (2020): 1675–1681.

[25] M. Kodama , T. Okimoto , K. Mizukami , et al., “Evaluation of a Novel Anti‐ *H. pylori* Antibody Detection Kit by Latex Turbidimetric Immunoassay,” Clinical Laboratory 65 (2019): 929–936, 10.7754/Clin.Lab.2018.180918. 31232019

[26] S. Kawai , K. Arai , Y. Lin , et al., “Comparison of the Detection of *Helicobacter pylori* Infection by Commercially Available Serological Testing Kits and the 13C‐Urea Breath Test,” Journal of Infection and Chemotherapy 25, no. 10 (2019): 769–773, 10.1016/j.jiac.2019.03.026. 31023569

[27] K. Tsutsumi , C. Kusano , S. Suzuki , T. Gotoda , and K. Murakami , “Diagnostic Accuracy of Latex Agglutination Turbidimetric Immunoassay in Screening Adolescents for *Helicobacter pylori* Infection in Japan,” Digestion 98, no. 2 (2018): 75–80, 10.1159/000487184. 29698942

[28] M. Miura , M. Ohtaka , M. Hanawa , et al., “Infection Status of *Helicobacter pylori* With Antibody Values of 3.0 U/mL or More and Less Than 10.0 U/mL in the LZ Test “Eiken” *H. pylori* Antibody,” Nihon Shokakibyo Gakkai Zasshi 115, no. 8 (2018): 721–731, 10.11405/nisshoshi.115.721. 30101873

[29] T. Kawai , M. Ito , N. Aoyama , et al., “Evaluation of Gastric Cancer Risk by Optimized Serum Antibody Titers Against *H. pylori* : A Multi‐Center Retrospective Study,” Journal of Japan Society for Helicobacter Research 19 (2018): 133–138.

[30] C. Kobayashi , Y. Nakanishi , and K. Murabayashi , “Clinical Evaluation of Sphere Light *H. pylori* Antibody J in the Diagnosis Before *H. pylori* Eradication,” Nisseki Kensa 50 (2017): 36–41.

[31] K. Kaihara , K. Miyake , K. Komatsu , A. Ebihara , and K. Nakayama , “Evaluating the Diagnostic Utility of a Serum Anti‐ *Helicobacter pylori* Antibody Assay Using a Latex Agglutination Turbidimetric Immunoassay,” Rinsho Byori 65, no. 7 (2017): 773–777.

[32] M. Inui , S. Ohwada , Y. Inui , and Y. Kondo , “Analysis of the Gray‐Zone Cutoff Values of New Serum *Helicobacter pylori* Antibody Kits Using Latex Immunoassay,” Nihon Shokakibyo Gakkai Zasshi 114, no. 11 (2017): 1968–1977, 10.11405/nisshoshi.114.1968. 29109345

[33] N. Aoyama , S. Shigeta , and H. Yokozaki , “Comparison of *H. pylori* Antibody Between E‐Plate (ELISA) and Latex Agglutination Method (LATEX) Among Strictly Diagnosed *H. pylori* Infection Status,” Journal of Japan Society for Helicobacter Research 18 (2017): 4–11.

[34] T. Furuta , “Evaluation of the Novel Anti‐ *H. pylori* Antibody Detection Kit for a Clinical Chemistry Auto‐Analyzer,” Japanese Journal of Medical Science 73 (2016): 451–457.

[35] H. Osumi , J. Fujisaki , T. Tsuchida , et al., “Clinical Trial of Evaluation of New Anti‐ *Helicobacter pylori* Antibody Using Latex Method,” Japanese Journal of Medical Science 73 (2016): 615–620.

[36] M. Kodama , T. Okimoto , R. Ogawa , K. Okamoto , K. Mizukami , and K. Murakami , “Evaluation of the Usefulness of Novel Anti‐ *H. pylori* Antibody Detection Kit Using a Latex Turbidimetric Immunoassay,” Japanese Journal of Medical Science 73 (2016): 737–744.

[37] M. Shiwa , M. Hirai , M. Horioka , et al., “Evaluation of a *Helicobacter pylori* Antibody Test Based on a Novel Japanese Strain Isolated From a Japanese Patient,” Japanese Journal of Clinical Laboratory Automation 41 (2016): 285–292.

[38] M. Inui , S. Ohwada , F. Ishida , and S.‐E. Kudo , “Serum *Helicobacter pylori* IgG Titers Are Predictive of *H. pylori* Infection Status,” Showa University Journal of Medical Sciences 28, no. 3 (2016): 233–240, 10.15369/sujms.28.233.

[39] M. Inui , S. Ohwada , Y. Inui , Y. Kondo , and K. Fukuda , “Diagnostic Accuracy of a New Serum *Helicobacter pylori* Antibody Detection Kit Using Latex Immunoassay,” Japanese Journal of Medical and Pharmaceutical Science 73 (2016): 335–338.

[40] M. Inui , S. Ohwada , Y. Inui , Y. Kondo , K. Fukuda , and Y. Matsumoto , “Comparative Analysis of *Helicobacter pylori* Infection Status Using Four Serum *Helicobacter pylori* Antibody Detection Kits in the Same Serum Sample,” Helicobacter Research 20 (2016): 621–627.

[41] K. Inoue , N. Maeda , T. Yoshioka , Y. Okamoto , and K. Takahashi , “Evaluation of “LZ Test EIKEN *H. pylori* Antibody” for the Diagnosis of *Helicobacter pylori* Infection,” Japanese Journal of Medical and Pharmaceutical Science 72 (2015): 331–337.

[42] M. Kodama , T. Okimoto , R. Ogawa , K. Okamoto , K. Mizukami , and K. Murakami , “Evaluation of the Usefulness of Novel EIA Kit for Anti‐ *H. pylori* IgG Antibody,” Japanese Journal of Medical and Pharmaceutical Science 72 (2015): 753–759.

[43] M. Inui , S. Ohwada , Y. Inui , Y. Kondo , N. Sohara , and K. Fukuda , “Clinical Evaluation of Serum Anti‐ *Helicobacter pylori* IgG Antibody Kits in Clinical Practice,” Helicobacter Research 19 (2015): 400–404.

[44] T. Furuta and M. Sugimoto , “Evaluation of the Novel Enzyme Immunoassay Detection Kit for Anti‐ *H. pylori* IgG Antibody,” Japanese Journal of Medical and Pharmaceutical Science 72 (2015): 325–330.

[45] J. Ueda , M. Okuda , T. Nishiyama , Y. Lin , Y. Fukuda , and S. Kikuchi , “Diagnostic Accuracy of the E‐Plate Serum Antibody Test Kit in Detecting *Helicobacter pylori* Infection Among Japanese Children,” Journal of Epidemiology 24, no. 1 (2014): 47–51, 10.2188/jea.JE20130078. 24240631 PMC3872524

[46] C. Iino , T. Shimoyama , T. Oyama , et al., “Evaluation of the Appropriate Cut‐Off Value for Serological Diagnosis of *Helicobacter pylori* Infection by Comparison With a Stool Antigen Test,” Hirosaki Igaku 63 (2013): 48–54.

[47] T. Matsuhisa and N. Yamada , “Efficacy of Determiner *H. pylori* J in the Diagnosis of *Helicobacter pylori* Infection,” Japanese Journal of Medical and Pharmaceutical Science 55 (2006): 429–435.

[48] M. Okuda , T. Sugiyama , K. Fukunaga , M. Kondou , E. Miyashiro , and T. Nakazawa , “A Strain‐Specific Antigen in Japanese *Helicobacter pylori* Recognized in Sera of Japanese Children,” Clinical and Diagnostic Laboratory Immunology 12, no. 11 (2005): 1280–1284, 10.1128/CDLI.12.11.1280-1284.2005. 16275941 PMC1287759

[49] K. Mabe , S. Kikuchi , M. Okuda , M. Takamasa , M. Kato , and M. Asaka , “Diagnostic Accuracy of Urine *Helicobacter pylori* Antibody Test in Junior and Senior High School Students in Japan,” Helicobacter 22, no. 1 (2017): e12329, 10.1111/hel.12329. 27400382

[50] K. Inoue , K. Fukushima , K. Mabe , T. Kamata , and K. Haruma , “Prospective Study About the Accuracy of Urinary *Helicobacter pylori* Antibody in Young Adults,” J Gastroenterol Cancer Screen 55 (2017): 340–348.

[51] M. Okuda , K. Mabe , Y. Lin , et al., “Rapid Urine Antibody Test for *Helicobacter pylori* Infection in Adolescents,” Pediatrics International 59, no. 7 (2017): 798–802, 10.1111/ped.13286. 28371166

[52] T. Shimoyama , Y. Sawada , N. Sawada , D. Chinda , and S. Fukuda , “Accuracy of a Stick‐Type Kit and Enzyme‐Linked Immunosorbent Assay in Detecting *Helicobacter pylori* Antibodies in Urine of People Living in the Japan Sea Region of Northern Japan,” Japanese Journal of Infectious Diseases 70, no. 2 (2017): 207–209, 10.7883/yoken.JJID.2015.642. 27357986

[53] M. Okuda , S. Kamiya , M. Booka , et al., “Diagnostic Accuracy of Urine‐Based Kits for Detection of *Helicobacter pylori* Antibody in Children,” Pediatrics International 55, no. 3 (2013): 337–341, 10.1111/ped.12057. 23360308

[54] M. Okuda , T. Nakazawa , M. Booka , E. Miyashiro , and N. Yosikawa , “Evaluation of a Urine Antibody Test for *Helicobacter pylori* in Japanese Children,” Journal of Pediatrics 144, no. 2 (2004): 196–199, 10.1016/j.jpeds.2003.10.057. 14760261

[55] K. Kubo , K. Mabe , S. Kikuchi , and M. Kato , “Diagnostic Accuracy of a Novel Stool Antigen Test for *Helicobacter pylori* Infection in a Medical Checkup Setting: A Prospective Cohort Study,” Internal Medicine 63, no. 11 (2024): 1525–1529, 10.2169/internalmedicine.2412-23. 37926549 PMC11189694

[56] T. Furuta and N. Shirai , “Clinical Evaluation of “DK14‐HP‐001”, *Helicobacter pylori* Antigen Kit, Using Stool Specimens,” Japanese Journal of Medical Science 79 (2022): 505–515.

[57] S. Ito , M. Ito , T. Chiba , T. Tadano , D. Shibuya , and K. Kato , “Evaluation of BLEIA EIKEN *H. pylori* Antigen Dedicated for Automated Bioluminescent Enzyme Immunoassay Analyzer BLEIA‐1200,” Japanese Journal of Medical Science 77 (2020): 1077–1086.

[58] M. Okuda , T. Osaki , S. Kikuchi , et al., “Evaluation of a Stool Antigen Test Using a mAb for Native Catalase for Diagnosis of *Helicobacter pylori* Infection in Children and Adults,” Journal of Medical Microbiology 63, no. 12 (2014): 1621–1625, 10.1099/jmm.0.077370-0. 25332372

[59] N. Sakaedani , M. Okuda , J. Kotake , et al., “Evaluation of the Usefulness of Premier Platinum HpSA Plus, a Stool Antigen Detection Kit Using Monoclonal Antibody,” Helicobacter Research 13 (2009): 415–419.

[60] K. Asahi , H. Yamanishi , and S. Kawano , “Evaluation of a *Helicobacter pylori* Stool Antigen Test With Monoclonal Antibody Specific for *H. pylori* ,” Japanese Journal of Medical and Pharmaceutical Science 57 (2007): 253–260.

[61] T. Sakamoto , H. Kato , R. Yamada , C. Tsunoda , M. Aiba , and S. Haga , “Comparison of a Monoclonal Stool Antigen Test and a Rapid Urease Test in the Diagnosis of *Helicobacter pylori* Infection,” Tokyo Joshi Ika Daigaku Zasshi 77 (2007): 331–336.

[62] M. Koike , H. Tajiri , M. Okuda , et al., “Evaluation of Novel Stool Antigen Tests for the Diagnosis of *Helicobacter pylori* Infection in Children: Comparison With 13C‐Urea Breath Test and Relation Between *Helicobacter pylori* Infection and Iron Deficiency Anemia of Childhood,” Journal of Japan Society for Helicobacter Research 7 (2005): 1–12.

[63] C. Wang , T. Nishiyama , S. Kikuchi , et al., “Changing Trends in the Prevalence of *H. pylori* Infection in Japan (1908–2003): A Systematic Review and Meta‐Regression Analysis of 170,752 Individuals,” Scientific Reports 7, no. 1 (2017): 15491, 10.1038/s41598-017-15490-7. 29138514 PMC5686167

[64] F. Ishibashi , C. Ichita , A. Takasu , et al., “The Increasing Trend of the Generational *Helicobacter pylori* ‐naïve Prevalence Among Japanese Individuals Born Between 1925 and 2015: A Systematic Review and Meta‐Regression Analysis,” Helicobacter 30, no. 3 (2025): e70052, 10.1111/hel.70052. 40500921

[65] C. Ichita , C. Kusano , T. Gotoda , et al., “Prevalence and Incidence of Gastric Cancer Among *Helicobacter pylori* ‐naïve Individuals in Japan: A Systematic Review and Meta‐Analysis,” Journal of Gastroenterology and Hepatology 41, no. 2 (2026): 454–464, 10.1111/jgh.70217. 41462573

[66] O. Toyoshima , T. Nishizawa , M. Arita , et al., “ *Helicobacter pylori* Infection in Subjects Negative for High Titer Serum Antibody,” World Journal of Gastroenterology 24, no. 13 (2018): 1419–1428, 10.3748/wjg.v24.i13.1419. 29632423 PMC5889822

[67] M. Inoue , N. Sawada , A. Goto , et al., “High‐Negative Anti‐ *Helicobacter pylori* IgG Antibody Titers and Long‐Term Risk of Gastric Cancer: Results From a Large‐Scale Population‐Based Cohort Study in Japan,” Cancer Epidemiology, Biomarkers & Prevention 29, no. 2 (2020): 420–426, 10.1158/1055-9965.EPI-19-0993. 31826914

