# Supplementary material for: Diagnostic Accuracy of Serum Antibody, Urine Antibody, and Stool Antigen Tests for Helicobacter pylori in Japan: A Systematic Review and Meta‐Analysis
Source: Helicobacter. 2026 Aug 16;31(4):e70157. doi: 10.1111/hel.70157 (PMC13477768; doi:10.1111/hel.70157)
Supplement: Supplementary file 1 [file HEL-31-e70157-s001.docx]

**Supplementary Materials**

**SUPPLEMENTARY TABLES**

**Supplementary Table 1. Search strategy**

**Supplementary Table 2. 2 × 2 contingency data for all included study–test combinations**

**Supplementary Table 3. Quality assessment of included studies using QUADAS-2**

**Supplementary Table 4. Subgroup analysis by presence of previously infected individuals**

**Supplementary Table 5. Subgroup analysis: post-hoc cutoff use**

**Supplementary Table 6a. Subgroup analysis: test kit type**

**Supplementary Table 6b. Subgroup analysis of serum antibody tests by test kit type after stratification by the presence of previously infected individuals**

**Supplementary Table 6c. Additional analysis of stool antigen EIA tests after separating BLEIA and excluding polyclonal HpSA**

**Supplementary Table 7. Subgroup analysis: age group**

**Supplementary Table 8. Subgroup analysis: reference standard composition**

**Supplementary Table 9. Analysis under clinically relevant conditions**

**SUPPLEMENTARY FIGURE LEGENDS**

**Supplementary** **Figure 1. Summary ROC curves of serum antibody tests.**

**Supplementary Figure 2. Summary ROC curve of urine antibody tests.**

**Supplementary Figure 3. Summary ROC curves of stool antigen tests.**

**Supplementary Figure 4. Forest plots: serum antibody, studies with previously infected individuals**

**Supplementary Figure 5. Forest plots: serum antibody, studies without previously infected individuals**

**Supplementary Figure 6. Forest plots: serum antibody, without post-hoc cutoff use**

**Supplementary Figure 7. Forest plots: serum antibody, with post-hoc cutoff use**

**Supplementary Figure 8. Forest plots: serum antibody, EIA (E-plate)**

**Supplementary Figure 9. Forest plots: serum antibody, EIA (non-E-plate)**

**Supplementary Figure 10. Forest plots: serum antibody, PA/LA**

**Supplementary Figure 11. Forest plots: urine antibody, EIA**

**Supplementary Figure 12. Forest plots: urine antibody, ICA**

**Supplementary Figure 13. Forest plots: stool antigen, EIA**

**Supplementary Figure 14. Forest plots: serum antibody, adults**

**Supplementary Figure 15. Forest plots: serum antibody, children**

**Supplementary Figure 16. Forest plots: urine antibody, adults**

**Supplementary Figure 17. Forest plots: urine antibody, children**

**Supplementary Figure 18. Forest plots: stool antigen, adults**

**Supplementary Figure 19. Forest plots: stool antigen, children**

**Supplementary Figure 20. Forest plots: serum antibody, composite reference standards**

**Supplementary Figure 21. Forest plots: serum antibody, single reference standard**

**Supplementary Figure 22. Forest plots: urine antibody, composite reference standards**

**Supplementary Figure 23. Forest plots: urine antibody, single reference standard**

**Supplementary Figure 24. Forest plots: stool antigen, composite reference standards**

**Supplementary Figure 25. Forest plots: stool antigen, single reference standard**

**Supplementary Figure 26. Forest plots: serum antibody, clinically relevant conditions**

**Supplementary Figure 27. SROC curve: serum antibody, clinically relevant conditions**

**Supplementary Figure 28. Forest plots: urine antibody, clinically relevant conditions**

**Supplementary Figure 29. SROC curve: urine antibody, clinically relevant conditions**

**Supplementary Figure 30. Forest plots: stool antigen, clinically relevant conditions**

**Supplementary Table 1. Search strategy**

| **Database** | **Search strategy** |
| --- | --- |
| **PubMed**  Search date:  January 20, 2025 | #1 Helicobacter pylori[MeSH Terms] OR helicobacter pylori[tw] OR h pylori[tw] OR h. pylori[tw] OR pylori[tw]  #2 ("Rapid Urease"[tw] OR "Urea Breath Test"[tw] OR histology[tw] OR culture[tw] OR serology[tw] OR "Stool Antigen Test"[tw] OR "Polymerase Chain Reaction"[tw] OR PCR[tw] OR "Diagnostic Test"[tw] OR antibody[tw] OR IgG[tw] OR ELISA[tw])  #3 ("Sensitivity and Specificity"[MeSH Terms] OR sensitivity[tw] OR specificity[tw] OR "Diagnostic Accuracy"[tw] OR accuracy[tw] OR "Predictive Value"[tw] OR "False Positive"[tw] OR "False Negative"[tw] OR "Likelihood Ratio"[tw] OR "Area Under Curve"[tw] OR AUC[tw] OR ROC[tw] OR "Receiver Operating Characteristic"[tw])  #4 "Japan"[tw] OR "Japanese"[tw]  #5 #1 AND #2 AND #3 AND #4  Filters: Publication date from 2005/01/01 to 2024/12/31 |
| **Embase**  Search date:  January 20, 2025 | #1 'Helicobacter pylori'/exp OR 'helicobacter pylori':ti,ab,kw OR 'h pylori':ti,ab,kw OR 'h. pylori':ti,ab,kw OR 'pylori':ti,ab,kw  #2 'rapid urease test'/exp OR 'rapid urease':ti,ab,kw OR 'urea breath test'/exp OR 'urea breath test':ti,ab,kw OR 'histology'/exp OR 'histology':ti,ab,kw OR 'culture'/exp OR 'culture':ti,ab,kw OR 'serology'/exp OR 'serology':ti,ab,kw OR 'stool antigen test':ti,ab,kw OR 'polymerase chain reaction'/exp OR 'polymerase chain reaction':ti,ab,kw OR 'PCR':ti,ab,kw OR 'diagnostic test'/exp OR 'diagnostic test':ti,ab,kw OR 'antibody':ti,ab,kw OR 'IgG':ti,ab,kw OR 'enzyme linked immunosorbent assay'/exp OR 'ELISA':ti,ab,kw  #3 'sensitivity and specificity'/exp OR 'sensitivity':ti,ab,kw OR 'specificity':ti,ab,kw OR 'diagnostic accuracy'/exp OR 'diagnostic accuracy':ti,ab,kw OR 'accuracy':ti,ab,kw OR 'predictive value'/exp OR 'predictive value':ti,ab,kw OR 'false positive':ti,ab,kw OR 'false negative':ti,ab,kw OR 'likelihood ratio':ti,ab,kw OR 'area under the curve'/exp OR 'area under curve':ti,ab,kw OR 'AUC':ti,ab,kw OR 'ROC':ti,ab,kw OR 'receiver operating characteristic'/exp OR 'receiver operating characteristic':ti,ab,kw  #4 'Japan'/exp OR 'japan':ti,ab,kw OR 'japanese':ti,ab,kw  #5 #1 AND #2 AND #3 AND #4  Filters: [2005-2024]/py AND [humans]/lim AND [embase]/lim NOT ([embase]/lim AND [medline]/lim) |
| **Ichushi-Web**  (Japan Medical  Abstracts Society)  Search date:  May 27, 2025 | #1 ("ピロリ"/AL *[Pylori]* or "ピロリ菌"/AL *[Pylori bacteria]* or "H. pylori"/AL or "H pylori"/AL or "pylori"/AL or "Hp"/AL or "ヘリコバクター"/AL *[Helicobacter]* or "ヘリコバクター・ピロリ"/AL *[Helicobacter pylori]* or "ヘリコバクターピロリ"/AL *[Helicobacter pylori]* or "Helicobacter"/AL or "Helicobacter pylori"/AL or "Helicobacter pylori"/TH)  and  #2 ("迅速ウレアーゼ"/AL *[Rapid urease]* or "迅速ウレアーゼ試験"/AL *[Rapid urease test]* or "RUT"/AL or "尿素呼気"/AL *[Urea breath]* or "尿素呼気試験"/AL *[Urea breath test]* or "尿素呼気テスト"/AL *[Urea breath test]* or "UBT"/AL or "組織"/AL *[Histology]* or "組織診"/AL *[Histological examination]* or "生検"/AL *[Biopsy]* or "培養"/AL *[Culture]* or "抗体"/AL *[Antibody]* or "抗体検査"/AL *[Antibody test]* or "血清抗体"/AL *[Serum antibody]* or "IgG"/AL or "便中抗原"/AL *[Stool antigen]* or "便中抗原検査"/AL *[Stool antigen test]* or "PCR"/AL or "ポリメラーゼ連鎖反応"/AL *[Polymerase chain reaction]* or "PCR法"/TH or "ELISA"/AL or "酵素免疫測定法"/AL *[Enzyme immunoassay]* or "酵素結合免疫吸着測定法"/AL *[Enzyme-linked immunosorbent assay]* or "酵素抗体法"/AL *[Enzyme antibody method]* or "酵素免疫法"/AL *[Enzyme immunoassay]* or "酵素結合免疫吸着法"/AL *[ELISA]* or "酵素結合免疫法"/AL *[Enzyme-linked immunoassay]* or "酵素抗体測定法"/AL *[Enzyme antibody assay]* or "EIA"/AL or "診断法"/AL *[Diagnostic method]* or "検査法"/AL) *[Test method]*  and  #3 ("感度"/AL *[Sensitivity]* or "特異度"/AL *[Specificity]* or "診断精度"/AL *[Diagnostic accuracy]* or "正診率"/AL *[Accuracy rate]* or "偽陽性"/AL *[False positive]* or "偽陰性"/AL *[False negative]* or "予測値"/AL *[Predictive value]* or "陽性的中率"/AL *[Positive predictive value]* or "陰性的中率"/AL *[Negative predictive value]* or "ROC"/AL or "受信者動作特性曲線"/AL *[Receiver operating characteristic curve]* or "Receiver Operating Characteristic"/AL or "ROC曲線"/TH or "AUC"/AL or "曲線下面積"/AL *[Area under the curve]* or "Area Under the Curve"/AL or "曲線下面積"/TH)  and  #4 ((FT=Y) DT=2005:2024 LA=日本語,英語 *[Language: Japanese, English]* (PT=症例報告・事例除く) *[Excluding: case reports]* AND (PT=原著論文) *[Article type: original articles]* CK=ヒト) *[Check tag: human]*  #1 AND #2 AND #3 AND #4  Search period: January 1, 2005 to December 31, 2024 |

For Ichushi-Web, original Japanese search terms are shown in bold, with English translations in *[italicized brackets]*.

**Supplementary Table 2. 2 × 2 contingency data for all included study–test combinations**

A. **Serum antibody**

| **No.** | **Author** | **Year** | **Index test (detail)** | **Test category** | **Cutoff** | **Unit** | **Post-hoc cutoff** | **Reference standard** | **Diagnostic objective** | **N** | **TP** | **FP** | **FN** | **TN** |
| --- | --- | --- | --- | --- | --- | --- | --- | --- | --- | --- | --- | --- | --- | --- |
| 1 | Inoue Kazuhiko | 2023 | L-type Wako H. pylori antibody J (CLEIA) | Serum PA/LA | 4 | U/mL | No | UBT and upper GI endoscopy | Current vs Others | 207 | 102 | 1 | 1 | 103 |
| 1 | Inoue Kazuhiko | 2023 | Latex Seiken H. pylori antibody (Denka LIA) | Serum PA/LA | 10 | U/mL | No | UBT and upper GI endoscopy | Current vs Others | 207 | 102 | 1 | 1 | 103 |
| 1 | Inoue Kazuhiko | 2023 | LZ test Eiken H. pylori antibody (LIA) | Serum PA/LA | 10 | U/mL | No | UBT and upper GI endoscopy | Current vs Others | 207 | 84 | 2 | 19 | 102 |
| 1 | Inoue Kazuhiko | 2023 | E-plate Eiken H. pylori antibody II (ELISA) | Serum EIA (E-plate) | 10 | U/mL | No | UBT and upper GI endoscopy | Current vs Others | 207 | 99 | 0 | 4 | 104 |
| 1 | Inoue Kazuhiko | 2023 | L-type Wako H. pylori antibody J (CLEIA) | Serum PA/LA | 4 | U/mL | No | UBT and upper GI endoscopy | Ever-infected vs Others | 207 | 103 | 1 | 1 | 102 |
| 1 | Inoue Kazuhiko | 2023 | Latex Seiken H. pylori antibody (Denka LIA) | Serum PA/LA | 10 | U/mL | No | UBT and upper GI endoscopy | Ever-infected vs Others | 207 | 103 | 1 | 1 | 102 |
| 1 | Inoue Kazuhiko | 2023 | LZ test Eiken H. pylori antibody (LIA) | Serum PA/LA | 10 | U/mL | No | UBT and upper GI endoscopy | Ever-infected vs Others | 207 | 102 | 19 | 2 | 84 |
| 1 | Inoue Kazuhiko | 2023 | E-plate Eiken H. pylori antibody II (ELISA) | Serum EIA (E-plate) | 10 | U/mL | No | UBT and upper GI endoscopy | Ever-infected vs Others | 207 | 104 | 4 | 0 | 99 |
| 2 | Takayama Takako | 2022 | LZ test (LIA) | Serum PA/LA | 10 | U/mL | No | SAT and endoscopic findings | Current vs Others | 482 | 33 | 13 | 5 | 431 |
| 2 | Takayama Takako | 2022 | LZ test (LIA) | Serum PA/LA | 10 | U/mL | No | SAT and endoscopic findings | Ever-infected vs Others | 482 | 409 | 27 | 5 | 41 |
| 3 | Kusano Chika | 2021 | E-plate Eiken H. pylori antibody (ELISA) | Serum EIA (E-plate) | 10 | U/mL | No | SAT | Current vs Others | 410 | 10 | 0 | 5 | 395 |
| 3 | Kusano Chika | 2021 | E-plate Eiken H. pylori antibody (ELISA) | Serum EIA (E-plate) | 10 | U/mL | No | SAT | Ever-infected vs Others | 410 | 395 | 5 | 0 | 10 |
| 4 | Ito Masanori | 2020 | LZ test (LIA) | Serum PA/LA | 3 | U/mL | No | UBT, RUT, SAT, and endoscopic atrophy | Ever-infected vs Others | 2622 | 1270 | 62 | 317 | 973 |
| 4 | Ito Masanori | 2020 | E-plate Eiken H. pylori antibody II (EIA) | Serum EIA (E-plate) | 3 | U/mL | No | UBT, RUT, SAT, and endoscopic atrophy | Ever-infected vs Others | 2622 | 1476 | 41 | 111 | 994 |
| 4 | Ito Masanori | 2020 | L-type Wako H. pylori antibody J (CLEIA) | Serum PA/LA | 4.4 | U/mL | Yes | UBT, RUT, SAT, and endoscopic atrophy | Ever-infected vs Others | 1521 | 743 | 81 | 39 | 658 |
| 4 | Ito Masanori | 2020 | H. pylori Latex Seiken (Denka LIA) | Serum PA/LA | 10.8 | U/mL | Yes | UBT, RUT, SAT, and endoscopic atrophy | Ever-infected vs Others | 1640 | 584 | 143 | 31 | 882 |
| 5 | Ueno Yusuke | 2020 | LZ test Eiken H. pylori antibody II (serum IgG, LIA) | Serum PA/LA | 5 | U/mL | No | Upper GI endoscopy and UBT | Current vs Others | 200 | 99 | 8 | 1 | 92 |
| 5 | Ueno Yusuke | 2020 | LZ test Eiken H. pylori antibody II (serum IgG, LIA) | Serum PA/LA | 5 | U/mL | No | Upper GI endoscopy and UBT | Ever-infected vs Others | 200 | 92 | 1 | 8 | 99 |
| 5 | Ueno Yusuke | 2020 | LZ test Eiken H. pylori antibody II (serum IgG, LIA) | Serum PA/LA | 5 | U/mL | No | Upper GI endoscopy and UBT | Ever-infected vs Others | 290 | 92 | 52 | 8 | 138 |
| 6 | Kodama Masaaki | 2019 | Denka H. pylori Latex Seiken (LIA) | Serum PA/LA | 10 | U/mL | No | Endoscopic gastric atrophy, RUT, and culture | Current vs Others | 152 | 71 | 5 | 6 | 70 |
| 6 | Kodama Masaaki | 2019 | Kit A: E-plate II Eiken (ELISA) | Serum EIA (E-plate) | 3.9 | U/mL | Yes | Endoscopic gastric atrophy, RUT, and culture | Current vs Others | 152 | 68 | 0 | 9 | 75 |
| 6 | Kodama Masaaki | 2019 | Kit B: Premier H. pylori kit (ELISA) | Serum EIA (non-E-plate) | 0.11 | U/mL | Yes | Endoscopic gastric atrophy, RUT, and culture | Current vs Others | 152 | 76 | 18 | 1 | 57 |
| 6 | Kodama Masaaki | 2019 | Kit B: Premier H. pylori kit (ELISA) | Serum EIA (non-E-plate) | 1.59 | U/mL | Yes | Endoscopic gastric atrophy, RUT, and culture | Current vs Others | 152 | 76 | 15 | 1 | 60 |
| 6 | Kodama Masaaki | 2019 | Denka H. pylori Latex Seiken (LIA) | Serum PA/LA | 10 | U/mL | No | Endoscopic gastric atrophy, RUT, and culture | Ever-infected vs Others | 152 | 70 | 6 | 5 | 71 |
| 6 | Kodama Masaaki | 2019 | Kit A: E-plate II Eiken (ELISA) | Serum EIA (E-plate) | 3.9 | U/mL | Yes | Endoscopic gastric atrophy, RUT, and culture | Ever-infected vs Others | 152 | 75 | 9 | 0 | 68 |
| 6 | Kodama Masaaki | 2019 | Kit B: Premier H. pylori kit (ELISA) | Serum EIA (non-E-plate) | 0.11 | U/mL | Yes | Endoscopic gastric atrophy, RUT, and culture | Ever-infected vs Others | 152 | 57 | 1 | 18 | 76 |
| 6 | Kodama Masaaki | 2019 | Kit B: Premier H. pylori kit (ELISA) | Serum EIA (non-E-plate) | 1.59 | U/mL | Yes | Endoscopic gastric atrophy, RUT, and culture | Ever-infected vs Others | 152 | 60 | 1 | 15 | 76 |
| 7 | Kawai Sayo | 2019 | E-plate Eiken H. pylori antibody II (ELISA) | Serum EIA (E-plate) | 10 | U/mL | No | UBT | Current vs Others | 200 | 150 | 1 | 4 | 45 |
| 7 | Kawai Sayo | 2019 | H. pylori IgG Seiken (EIA) | Serum EIA (non-E-plate) | 10 | U/mL | No | UBT | Current vs Others | 200 | 151 | 0 | 3 | 46 |
| 7 | Kawai Sayo | 2019 | IZ test Eiken H. pylori Antibody (IZ | Serum PA/LA | 10 | U/mL | No | UBT | Current vs Others | 200 | 153 | 2 | 1 | 44 |
| 7 | Kawai Sayo | 2019 | H. pylori Latex Seiken (LIA) | Serum PA/LA | 10 | U/mL | No | UBT | Current vs Others | 200 | 151 | 4 | 3 | 42 |
| 7 | Kawai Sayo | 2019 | E-plate Eiken H. pylori antibody II (ELISA) | Serum EIA (E-plate) | 10 | U/mL | No | UBT | Ever-infected vs Others | 200 | 45 | 4 | 1 | 150 |
| 7 | Kawai Sayo | 2019 | H. pylori IgG Seiken (EIA) | Serum EIA (non-E-plate) | 10 | U/mL | No | UBT | Ever-infected vs Others | 200 | 46 | 3 | 0 | 151 |
| 7 | Kawai Sayo | 2019 | IZ test Eiken H. pylori Antibody (IZ | Serum PA/LA | 10 | U/mL | No | UBT | Ever-infected vs Others | 200 | 44 | 1 | 2 | 153 |
| 7 | Kawai Sayo | 2019 | H. pylori Latex Seiken (LIA) | Serum PA/LA | 10 | U/mL | No | UBT | Ever-infected vs Others | 200 | 42 | 3 | 4 | 151 |
| 8 | Tsutsumi Koshiro | 2018 | LZ test (LIA) | Serum PA/LA | 3.1 | U/mL | Yes | SAT | Current vs Others | 209 | 7 | 17 | 1 | 184 |
| 8 | Tsutsumi Koshiro | 2018 | LZ test (LIA) | Serum PA/LA | 3.1 | U/mL | Yes | SAT | Ever-infected vs Others | 209 | 184 | 1 | 17 | 7 |
| 9 | Miura Mika | 2018 | LZ test Eiken H. pylori antibody (LIA) | Serum PA/LA | 5.6 | U/mL | Yes | UBT and endoscopic gastric atrophy | Current vs Others | 698 | 106 | 175 | 50 | 367 |
| 10 | Kawai Takashi | 2018 | E-plate serum H. pylori antibody (ELISA) | Serum EIA (E-plate) | 3 | U/mL | No | UBT, HpSA, RUT, and upper GI endoscopy | Ever-infected vs Others | 2591 | 582 | 59 | 32 | 1918 |
| 11 | Kobayashi Chiaki | 2017 | SphereLight H. pylori antibody J (CLEIA) | Serum EIA (non-E-plate) | 4 | U/mL | No | Composite: serum antibody (two methods), SAT, histology, and UBT | Current vs Others | 62 | 41 | 1 | 0 | 20 |
| 11 | Kobayashi Chiaki | 2017 | E-plate Eiken H. pylori antibody (ELISA) | Serum EIA (E-plate) | 10 | U/mL | No | Composite: serum antibody (two methods), SAT, histology, and UBT | Current vs Others | 62 | 34 | 3 | 7 | 18 |
| 11 | Kobayashi Chiaki | 2017 | SphereLight H. pylori antibody J (CLEIA) | Serum EIA (non-E-plate) | 4 | U/mL | No | Composite: serum antibody (two methods), SAT, histology, and UBT | Ever-infected vs Others | 62 | 20 | 0 | 1 | 41 |
| 11 | Kobayashi Chiaki | 2017 | E-plate Eiken H. pylori antibody (ELISA) | Serum EIA (E-plate) | 10 | U/mL | No | Composite: serum antibody (two methods), SAT, histology, and UBT | Ever-infected vs Others | 62 | 18 | 7 | 3 | 34 |
| 12 | Kaihara Kazumi | 2017 | LZ test (LIA) | Serum PA/LA | 10 | U/mL | No | UBT and upper GI endoscopy | Current vs Others | 116 | 38 | 7 | 7 | 64 |
| 12 | Kaihara Kazumi | 2017 | E-plate Eiken H. pylori antibody II (ELISA) | Serum EIA (E-plate) | 10 | U/mL | No | UBT and upper GI endoscopy | Current vs Others | 116 | 42 | 1 | 3 | 70 |
| 12 | Kaihara Kazumi | 2017 | LZ test (LIA) | Serum PA/LA | 10 | U/mL | No | UBT and upper GI endoscopy | Ever-infected vs Others | 116 | 64 | 7 | 7 | 38 |
| 12 | Kaihara Kazumi | 2017 | E-plate Eiken H. pylori antibody II (ELISA) | Serum EIA (E-plate) | 10 | U/mL | No | UBT and upper GI endoscopy | Ever-infected vs Others | 116 | 70 | 3 | 1 | 42 |
| 13 | Inui Masayuki | 2017 | Wako LIA (CLEIA) | Serum PA/LA | 4 | U/mL | No | Composite: endoscopy, RUT, UBT, and SAT | Current vs Others | 256 | 87 | 23 | 5 | 141 |
| 13 | Inui Masayuki | 2017 | Denka LIA | Serum PA/LA | 10 | U/mL | No | Composite: endoscopy, RUT, UBT, and SAT | Current vs Others | 256 | 88 | 16 | 4 | 148 |
| 13 | Inui Masayuki | 2017 | Eiken LZ (LIA) | Serum PA/LA | 10 | U/mL | No | Composite: endoscopy, RUT, UBT, and SAT | Current vs Others | 256 | 79 | 13 | 13 | 151 |
| 13 | Inui Masayuki | 2017 | Wako LIA (CLEIA) | Serum PA/LA | 4 | U/mL | No | Composite: endoscopy, RUT, UBT, and SAT | Ever-infected vs Others | 256 | 125 | 21 | 4 | 106 |
| 13 | Inui Masayuki | 2017 | Denka LIA | Serum PA/LA | 10 | U/mL | No | Composite: endoscopy, RUT, UBT, and SAT | Ever-infected vs Others | 256 | 125 | 27 | 4 | 100 |
| 13 | Inui Masayuki | 2017 | Eiken LZ (LIA) | Serum PA/LA | 10 | U/mL | No | Composite: endoscopy, RUT, UBT, and SAT | Ever-infected vs Others | 256 | 123 | 41 | 6 | 86 |
| 14 | Aoyama Nobuo | 2017 | E-plate (ELISA) | Serum EIA (E-plate) | 10 | U/mL | No | RUT, UBT, endoscopy, and histopathology | Current vs Others | 261 | 96 | 0 | 15 | 150 |
| 14 | Aoyama Nobuo | 2017 | LZ test (LIA) | Serum PA/LA | 10 | U/mL | No | RUT, UBT, endoscopy, and histopathology | Current vs Others | 261 | 97 | 4 | 14 | 146 |
| 14 | Aoyama Nobuo | 2017 | E-plate (ELISA) | Serum EIA (E-plate) | 10 | U/mL | No | RUT, UBT, endoscopy, and histopathology | Ever-infected vs Others | 261 | 150 | 15 | 0 | 96 |
| 14 | Aoyama Nobuo | 2017 | LZ test (LIA) | Serum PA/LA | 10 | U/mL | No | RUT, UBT, endoscopy, and histopathology | Ever-infected vs Others | 261 | 146 | 14 | 4 | 97 |
| 15 | Furuta Takahisa | 2016 | Novel latex turbidimetric IgG (prototype RD-11-04) | Serum PA/LA | 10 | U/mL | No | Endoscopic gastric atrophy (Kimura-Takemoto) and RUT | Current vs Others | 228 | 203 | 1 | 13 | 11 |
| 15 | Furuta Takahisa | 2016 | Conventional latex (LIA) | Serum PA/LA | 10 | U/mL | No | Endoscopic gastric atrophy (Kimura-Takemoto) and RUT | Current vs Others | 228 | 186 | 1 | 30 | 11 |
| 15 | Furuta Takahisa | 2016 | Conventional EIA | Serum EIA (E-plate) | 10 | U/mL | No | Endoscopic gastric atrophy (Kimura-Takemoto) and RUT | Current vs Others | 228 | 194 | 0 | 22 | 12 |
| 15 | Furuta Takahisa | 2016 | Novel latex turbidimetric IgG (prototype RD-11-04) | Serum PA/LA | 10 | U/mL | No | Endoscopic gastric atrophy (Kimura-Takemoto) and RUT | Ever-infected vs Others | 228 | 11 | 13 | 1 | 203 |
| 15 | Furuta Takahisa | 2016 | Conventional latex (LIA) | Serum PA/LA | 10 | U/mL | No | Endoscopic gastric atrophy (Kimura-Takemoto) and RUT | Ever-infected vs Others | 228 | 11 | 30 | 1 | 186 |
| 15 | Furuta Takahisa | 2016 | Conventional EIA | Serum EIA (E-plate) | 10 | U/mL | No | Endoscopic gastric atrophy (Kimura-Takemoto) and RUT | Ever-infected vs Others | 228 | 12 | 22 | 0 | 194 |
| 16 | Osumi Hiroki | 2016 | LZ serum antibody (LIA) | Serum PA/LA | 10 | U/mL | No | UBT | Current vs Others | 205 | 70 | 18 | 10 | 107 |
| 16 | Osumi Hiroki | 2016 | E-plate Eiken (ELISA) | Serum EIA (E-plate) | 10 | U/mL | No | UBT | Current vs Others | 205 | 72 | 1 | 8 | 124 |
| 16 | Osumi Hiroki | 2016 | LZ serum antibody (LIA) | Serum PA/LA | 10 | U/mL | No | UBT | Ever-infected vs Others | 205 | 107 | 10 | 18 | 70 |
| 16 | Osumi Hiroki | 2016 | E-plate Eiken (ELISA) | Serum EIA (E-plate) | 10 | U/mL | No | UBT | Ever-infected vs Others | 205 | 124 | 8 | 1 | 72 |
| 17 | Kodama Masaaki | 2016 | Novel Denka latex kit (LIA) | Serum PA/LA | 10 | U/mL | No | Endoscopic atrophy, RUT, and culture | Current vs Others | 101 | 47 | 3 | 3 | 48 |
| 17 | Kodama Masaaki | 2016 | LZ test (LIA) | Serum PA/LA | 10 | U/mL | No | Endoscopic atrophy, RUT, and culture | Current vs Others | 101 | 46 | 1 | 4 | 50 |
| 17 | Kodama Masaaki | 2016 | E-plate (ELISA) | Serum EIA (E-plate) | 10 | U/mL | No | Endoscopic atrophy, RUT, and culture | Current vs Others | 101 | 47 | 0 | 3 | 51 |
| 17 | Kodama Masaaki | 2016 | Novel Denka latex kit (LIA) | Serum PA/LA | 10 | U/mL | No | Endoscopic atrophy, RUT, and culture | Ever-infected vs Others | 101 | 48 | 3 | 3 | 47 |
| 17 | Kodama Masaaki | 2016 | LZ test (LIA) | Serum PA/LA | 10 | U/mL | No | Endoscopic atrophy, RUT, and culture | Ever-infected vs Others | 101 | 50 | 4 | 1 | 46 |
| 17 | Kodama Masaaki | 2016 | E-plate (ELISA) | Serum EIA (E-plate) | 10 | U/mL | No | Endoscopic atrophy, RUT, and culture | Ever-infected vs Others | 101 | 51 | 3 | 0 | 47 |
| 18 | Shiwa Masaaki | 2016 | SphereLight H. pylori antibody J (CLEIA) | Serum EIA (non-E-plate) | 4 | U/mL | No | Histological examination | Current vs Others | 100 | 48 | 0 | 1 | 51 |
| 18 | Shiwa Masaaki | 2016 | E-plate Eiken H. pylori antibody II (ELISA) | Serum EIA (E-plate) | 10 | U/mL | No | Histological examination | Current vs Others | 100 | 44 | 0 | 5 | 51 |
| 18 | Shiwa Masaaki | 2016 | SphereLight H. pylori antibody J (CLEIA) | Serum EIA (non-E-plate) | 4 | U/mL | No | Histological examination | Ever-infected vs Others | 100 | 51 | 1 | 0 | 48 |
| 18 | Shiwa Masaaki | 2016 | E-plate Eiken H. pylori antibody II (ELISA) | Serum EIA (E-plate) | 10 | U/mL | No | Histological examination | Ever-infected vs Others | 100 | 51 | 5 | 0 | 44 |
| 21 | Inui Masayuki | 2016 | SphereLight H. pylori IgG J (CLEIA) | Serum EIA (non-E-plate) | 4 | U/mL | No | Composite: RUT, UBT, monoclonal SAT, or histology, combined with endoscopy | Current vs Others | 273 | 78 | 22 | 5 | 168 |
| 21 | Inui Masayuki | 2016 | Wako LIA-HP serum IgG antibody (CLEIA) | Serum PA/LA | 4 | U/mL | No | Composite: RUT, UBT, SAT, urine antibody, and endoscopy | Current vs Others | 142 | 54 | 2 | 2 | 84 |
| 21 | Inui Masayuki | 2016 | L-type Wako H. pylori antibody J (CLEIA) | Serum PA/LA | 4 | U/mL | No | Upper GI endoscopy, RUT, UBT, and SAT | Current vs Others | 232 | 81 | 21 | 4 | 126 |
| 21 | Inui Masayuki | 2016 | SphereLight H. pylori antibody J (CLEIA) | Serum EIA (non-E-plate) | 4 | U/mL | No | Upper GI endoscopy, RUT, UBT, and SAT | Current vs Others | 232 | 80 | 21 | 5 | 126 |
| 21 | Inui Masayuki | 2016 | LZ test Eiken H. pylori antibody (LIA) | Serum PA/LA | 10 | U/mL | No | Upper GI endoscopy, RUT, UBT, and SAT | Current vs Others | 232 | 75 | 11 | 10 | 136 |
| 21 | Inui Masayuki | 2016 | E-plate Eiken H. pylori antibody II (ELISA) | Serum EIA (E-plate) | 10 | U/mL | No | Upper GI endoscopy, RUT, UBT, and SAT | Current vs Others | 232 | 73 | 4 | 12 | 143 |
| 21 | Inui Masayuki | 2016 | SphereLight H. pylori IgG J (CLEIA) | Serum EIA (non-E-plate) | 4 | U/mL | No | Composite: RUT, UBT, monoclonal SAT, or histology, combined with endoscopy | Ever-infected vs Others | 273 | 129 | 44 | 5 | 95 |
| 21 | Inui Masayuki | 2016 | Wako LIA-HP serum IgG antibody (CLEIA) | Serum PA/LA | 4 | U/mL | No | Composite: RUT, UBT, SAT, urine antibody, and endoscopy | Ever-infected vs Others | 142 | 84 | 2 | 2 | 54 |
| 21 | Inui Masayuki | 2016 | L-type Wako H. pylori antibody J (CLEIA) | Serum PA/LA | 4 | U/mL | No | Upper GI endoscopy, RUT, UBT, and SAT | Ever-infected vs Others | 232 | 109 | 21 | 5 | 97 |
| 21 | Inui Masayuki | 2016 | SphereLight H. pylori antibody J (CLEIA) | Serum EIA (non-E-plate) | 4 | U/mL | No | Upper GI endoscopy, RUT, UBT, and SAT | Ever-infected vs Others | 232 | 108 | 23 | 6 | 95 |
| 21 | Inui Masayuki | 2016 | LZ test Eiken H. pylori antibody (LIA) | Serum PA/LA | 10 | U/mL | No | Upper GI endoscopy, RUT, UBT, and SAT | Ever-infected vs Others | 232 | 109 | 37 | 5 | 81 |
| 21 | Inui Masayuki | 2016 | E-plate Eiken H. pylori antibody II (ELISA) | Serum EIA (E-plate) | 10 | U/mL | No | Upper GI endoscopy, RUT, UBT, and SAT | Ever-infected vs Others | 232 | 114 | 41 | 0 | 77 |
| 22 | Inoue Kazuhiko | 2015 | LZ test (LIA) | Serum PA/LA | 10 | U/mL | No | E-plate and UBT | Current vs Others | 255 | 55 | 11 | 7 | 182 |
| 22 | Inoue Kazuhiko | 2015 | LZ test (LIA) | Serum PA/LA | 10 | U/mL | No | E-plate and UBT | Ever-infected vs Others | 255 | 182 | 7 | 11 | 55 |
| 23 | Kodama Masaaki | 2015 | Denka latex (LIA) | Serum PA/LA | 10 | U/mL | No | Endoscopic atrophy, RUT, and culture | Current vs Others | 102 | 49 | 0 | 1 | 52 |
| 23 | Kodama Masaaki | 2015 | Conventional EIA | Serum EIA (E-plate) | 10 | U/mL | No | Endoscopic atrophy, RUT, and culture | Current vs Others | 102 | 46 | 0 | 4 | 52 |
| 23 | Kodama Masaaki | 2015 | Denka latex (LIA) | Serum PA/LA | 10 | U/mL | No | Endoscopic atrophy, RUT, and culture | Ever-infected vs Others | 102 | 52 | 1 | 0 | 49 |
| 23 | Kodama Masaaki | 2015 | Conventional EIA | Serum EIA (E-plate) | 10 | U/mL | No | Endoscopic atrophy, RUT, and culture | Ever-infected vs Others | 102 | 52 | 4 | 0 | 46 |
| 24 | Inui Masayuki | 2015 | E-plate | Serum EIA (E-plate) | 10 | U/mL | No | Composite: RUT, UBT, urine antibody, SAT, endoscopy, and eradication history | Current vs Others | 92 | 15 | 4 | 2 | 71 |
| 24 | Inui Masayuki | 2015 | LZ | Serum PA/LA | 10 | U/mL | No | Composite: RUT, UBT, urine antibody, SAT, endoscopy, and eradication history | Current vs Others | 92 | 16 | 10 | 1 | 65 |
| 24 | Inui Masayuki | 2015 | SphereLight J (CLEIA) | Serum EIA (non-E-plate) | 4 | U/mL | No | Composite: RUT, UBT, urine antibody, SAT, endoscopy, and eradication history | Current vs Others | 92 | 16 | 11 | 1 | 64 |
| 24 | Inui Masayuki | 2015 | E-plate | Serum EIA (E-plate) | 10 | U/mL | No | Composite: RUT, UBT, urine antibody, SAT, endoscopy, and eradication history | Ever-infected vs Others | 92 | 15 | 0 | 25 | 52 |
| 24 | Inui Masayuki | 2015 | LZ | Serum PA/LA | 10 | U/mL | No | Composite: RUT, UBT, urine antibody, SAT, endoscopy, and eradication history | Ever-infected vs Others | 92 | 20 | 0 | 20 | 52 |
| 24 | Inui Masayuki | 2015 | SphereLight J (CLEIA) | Serum EIA (non-E-plate) | 4 | U/mL | No | Composite: RUT, UBT, urine antibody, SAT, endoscopy, and eradication history | Ever-infected vs Others | 92 | 19 | 3 | 21 | 49 |
| 25 | Furuta Takahisa | 2015 | Denka EIA IgG antibody kit (prototype) | Serum EIA (non-E-plate) | 10 | U/mL | No | Endoscopic gastric atrophy and RUT | Current vs Others | 190 | 170 | 1 | 10 | 9 |
| 25 | Furuta Takahisa | 2015 | Conventional serum anti-H. pylori IgG EIA kit | Serum EIA (E-plate) | 10 | U/mL | No | Endoscopic gastric atrophy and RUT | Current vs Others | 190 | 159 | 0 | 21 | 10 |
| 25 | Furuta Takahisa | 2015 | Denka EIA IgG antibody kit (prototype) | Serum EIA (non-E-plate) | 10 | U/mL | No | Endoscopic gastric atrophy and RUT | Ever-infected vs Others | 190 | 9 | 10 | 1 | 170 |
| 25 | Furuta Takahisa | 2015 | Conventional serum anti-H. pylori IgG EIA kit | Serum EIA (E-plate) | 10 | U/mL | No | Endoscopic gastric atrophy and RUT | Ever-infected vs Others | 190 | 10 | 21 | 0 | 159 |
| 26 | Ueda Junko | 2014 | E-plate Eiken serum IgG antibody (ELISA) | Serum EIA (E-plate) | 10 | U/mL | No | Stool antigen test (HpSA ELISA) | Current vs Others | 73 | 32 | 1 | 2 | 38 |
| 26 | Ueda Junko | 2014 | E-plate Eiken serum IgG antibody (ELISA) | Serum EIA (E-plate) | 10 | U/mL | No | Stool antigen test (HpSA ELISA) | Ever-infected vs Others | 73 | 38 | 2 | 1 | 32 |
| 27 | Iino Chikara | 2013 | E-plate IgG antibody (ELISA) | Serum EIA (E-plate) | 10 | U/mL | No | SAT (monoclonal ELISA; Testmate) | Current vs Others | 994 | 529 | 75 | 22 | 368 |
| 27 | Iino Chikara | 2013 | E-plate IgG antibody (ELISA) | Serum EIA (E-plate) | 10 | U/mL | No | SAT (monoclonal ELISA; Testmate) | Ever-infected vs Others | 994 | 368 | 22 | 75 | 529 |
| 28 | Matsuhisa Takeshi | 2006 | E-plate Eiken H. pylori antibody (ELISA) | Serum EIA (E-plate) | 10 | U/mL | No | Histopathology (H&E, Giemsa, immunohistochemistry) | Current vs Others | 80 | 68 | 0 | 4 | 8 |
| 28 | Matsuhisa Takeshi | 2006 | Determiner H. pylori antibody / HM-CAP (ELISA) | Serum EIA (non-E-plate) | Positive≥2.3,  Negative≤1.7 | EV | No | Histopathology (H&E, Giemsa, immunohistochemistry) | Current vs Others | 80 | 59 | 0 | 5 | 7 |
| 28 | Matsuhisa Takeshi | 2006 | Determiner H. pylori antibody J / JHM-CAP (ELISA) | Serum EIA (non-E-plate) | Positive≥2.3,  Negative≤1.7 | EV | No | Histopathology (H&E, Giemsa, immunohistochemistry) | Current vs Others | 80 | 68 | 0 | 2 | 6 |
| 28 | Matsuhisa Takeshi | 2006 | E-plate Eiken H. pylori antibody (ELISA) | Serum EIA (E-plate) | 10 | U/mL | No | Histopathology (H&E, Giemsa, immunohistochemistry) | Ever-infected vs Others | 80 | 8 | 4 | 0 | 68 |
| 28 | Matsuhisa Takeshi | 2006 | Determiner H. pylori antibody / HM-CAP (ELISA) | Serum EIA (non-E-plate) | Positive≥2.3,  Negative≤1.7 | EV | No | Histopathology (H&E, Giemsa, immunohistochemistry) | Ever-infected vs Others | 80 | 7 | 5 | 0 | 59 |
| 28 | Matsuhisa Takeshi | 2006 | Determiner H. pylori antibody J / JHM-CAP (ELISA) | Serum EIA (non-E-plate) | Positive≥2.3,  Negative≤1.7 | EV | No | Histopathology (H&E, Giemsa, immunohistochemistry) | Ever-infected vs Others | 80 | 6 | 2 | 0 | 68 |
| 29 | Okuda Masumi | 2005 | HM-CAP (EIA) | Serum EIA (non-E-plate) | Positive:  EIA value (EVs) ≥ 2.3 Equivocal: 1.8 ≤ EVs < 2.3  Negative: EVs < 1.8 2.3 | EVs | No | HpSA and UBT | Current vs Others | 131 | 40 | 4 | 3 | 84 |
| 29 | Okuda Masumi | 2005 | HM-CAP (EIA) | Serum EIA (non-E-plate) | Positive: EIA value (EVs) ≥ 2.3 Equivocal: 1.8 ≤ EVs < 2.3  Negative: EVs < 1.8 2.3 | EVs | No | HpSA and UBT | Current vs Others | 131 | 29 | 6 | 14 | 82 |
| 29 | Okuda Masumi | 2005 | HM-CAP (EIA) | Serum EIA (non-E-plate) | Positive: EIA value (EVs) ≥ 2.3 Equivocal: 1.8 ≤ EVs < 2.3  Negative: EVs < 1.8 2.3 | EVs | No | HpSA and UBT | Ever-infected vs Others | 131 | 84 | 3 | 4 | 40 |
| 29 | Okuda Masumi | 2005 | HM-CAP (EIA) | Serum EIA (non-E-plate) | Positive: EIA value (EVs) ≥ 2.3 Equivocal: 1.8 ≤ EVs < 2.3  Negative: EVs < 1.8 2.3 | EVs | No | HpSA and UBT | Ever-infected vs Others | 131 | 82 | 14 | 6 | 29 |

UBT, urea breath test; RUT, rapid urease test; SAT, stool antigen test; HpSA, Helicobacter pylori stool antigen; GI, gastrointestinal; ELISA, enzyme-linked immunosorbent assay; EIA, enzyme immunoassay; LIA, latex immunoassay; CLEIA, chemiluminescent enzyme immunoassay; ICA, immunochromatographic assay; PG, pepsinogen; PCR, polymerase chain reaction; H&E, hematoxylin and eosin.

B. **Urine antibody**

| **No.** | **Author** | **Year** | **Index test (detail)** | **Cutoff** | **Unit** | **Post-hoc cutoff** | **Reference standard** | **Diagnostic objective** | **N** | **TP** | **FP** | **FN** | **TN** |
| --- | --- | --- | --- | --- | --- | --- | --- | --- | --- | --- | --- | --- | --- |
| 30 | Mabe Katushiro | 2017 | URINELISA (Otsuka; ELISA) | 1 | CI | No | UBT, SAT, and serum antibody | Current vs Others | 780 | 40 | 26 | 1 | 713 |
| 30 | Mabe Katushiro | 2017 | URINELISA (Otsuka; ELISA) | 1 | CI | No | UBT, SAT, and serum antibody | Ever-infected vs Others | 780 | 713 | 1 | 26 | 40 |
| 31 | Inoue Kazuhiko | 2017 | RAPIRUN H. pylori antibody stick (ICA) | - | - | No | UBT | Current vs Others | 116 | 13 | 4 | 1 | 98 |
| 31 | Inoue Kazuhiko | 2017 | RAPIRUN H. pylori antibody stick (ICA) | - | - | No | UBT | Current vs Others | 77 | 8 | 4 | 2 | 63 |
| 31 | Inoue Kazuhiko | 2017 | URINELISA H. pylori antibody (ELISA) | 1 | CI | No | UBT | Current vs Others | 77 | 10 | 5 | 0 | 62 |
| 31 | Inoue Kazuhiko | 2017 | RAPIRUN H. pylori antibody stick (ICA) | - | - | No | UBT | Current vs Others | 77 | 9 | 3 | 2 | 63 |
| 31 | Inoue Kazuhiko | 2017 | URINELISA H. pylori antibody (ELISA) | 1 | CI | No | UBT | Current vs Others | 77 | 11 | 4 | 0 | 62 |
| 31 | Inoue Kazuhiko | 2017 | RAPIRUN H. pylori antibody stick (ICA) | - | - | No | UBT | Ever-infected vs Others | 116 | 98 | 1 | 4 | 13 |
| 31 | Inoue Kazuhiko | 2017 | RAPIRUN H. pylori antibody stick (ICA) | - | - | No | UBT | Ever-infected vs Others | 77 | 63 | 2 | 4 | 8 |
| 31 | Inoue Kazuhiko | 2017 | URINELISA H. pylori antibody (ELISA) | 1 | CI | No | UBT | Ever-infected vs Others | 77 | 62 | 0 | 5 | 10 |
| 31 | Inoue Kazuhiko | 2017 | RAPIRUN H. pylori antibody stick (ICA) | - | - | No | UBT | Ever-infected vs Others | 77 | 63 | 2 | 3 | 9 |
| 31 | Inoue Kazuhiko | 2017 | URINELISA H. pylori antibody (ELISA) | 1 | CI | No | UBT | Ever-infected vs Others | 77 | 62 | 0 | 4 | 11 |
| 32 | Okuda Masami | 2017 | Rapid urine H. pylori antibody (RAPIRUN; ICA) | - | - | No | Serum antibody ELISA | Current vs Others | 184 | 6 | 0 | 4 | 174 |
| 32 | Okuda Masami | 2017 | Urine H. pylori ELISA (u-HpELISA) | 1 | CI | No | Serum antibody ELISA | Ever-infected vs Others | 184 | 174 | 4 | 0 | 6 |
| 33 | Shimoyama Tadashi | 2017 | RAPIRUN (Otsuka; ICA) | - | - | No | UBT and monoclonal SAT | Current vs Others | 359 | 125 | 69 | 25 | 140 |
| 33 | Shimoyama Tadashi | 2017 | URINELISA (Otsuka; ELISA) | 1 | CI | No | UBT and monoclonal SAT | Current vs Others | 201 | 64 | 18 | 10 | 109 |
| 33 | Shimoyama Tadashi | 2017 | RAPIRUN (Otsuka; ICA) | - | - | No | UBT and monoclonal SAT | Ever-infected vs Others | 359 | 140 | 25 | 69 | 125 |
| 33 | Shimoyama Tadashi | 2017 | URINELISA (Otsuka; ELISA) | 1 | CI | No | UBT and monoclonal SAT | Ever-infected vs Others | 201 | 109 | 10 | 18 | 64 |
| 34 | Okuda Masumi | 2013 | URINELISA (Otsuka; ELISA) | 1 | CI | No | UBT and HpSA | Current vs Others | 101 | 34 | 2 | 3 | 62 |
| 34 | Okuda Masumi | 2013 | RAPIRUN (Otsuka; ICA) | - | - | No | UBT and HpSA | Current vs Others | 100 | 29 | 0 | 7 | 64 |
| 34 | Okuda Masumi | 2013 | URINELISA (Otsuka; ELISA) | 1 | CI | No | UBT and HpSA | Ever-infected vs Others | 101 | 62 | 3 | 2 | 34 |
| 34 | Okuda Masumi | 2013 | RAPIRUN (Otsuka; ICA) | - | - | No | No | Ever-infected vs Others | 100 | 64 | 7 | 0 | 29 |
| 35 | Okuda Masumi | 2004 | Urine H. pylori ELISA (URINELISA) | 1 | CI | No | UBT and HpSA | Current vs Others | 100 | 34 | 2 | 2 | 62 |
| 35 | Okuda Masumi | 2004 | Urine H. pylori ELISA (URINELISA) | 1 | CI | No | UBT and HpSA | Ever-infected vs Others | 100 | 62 | 2 | 2 | 34 |

UBT, urea breath test; RUT, rapid urease test; SAT, stool antigen test; HpSA, Helicobacter pylori stool antigen; GI, gastrointestinal; ELISA, enzyme-linked immunosorbent assay; EIA, enzyme immunoassay; LIA, latex immunoassay; CLEIA, chemiluminescent enzyme immunoassay; ICA, immunochromatographic assay; PG, pepsinogen; PCR, polymerase chain reaction; H&E, hematoxylin and eosin

C. **Stool antigen**

| **No.** | **Author** | **Year** | **Index test (detail)** | **Cutoff** | **Unit** | **Post-hoc cutoff** | **Reference standard** | **Diagnostic objective** | **N** | **TP** | **FP** | **FN** | **TN** |
| --- | --- | --- | --- | --- | --- | --- | --- | --- | --- | --- | --- | --- | --- |
| 36 | Kubo Kimitoshi | 2024 | Eiken H. pylori antigen kit (EIA) | 1 | COI | No | Endoscopy, eradication history, serum antibody, and serum PG II | Current vs Others | 1055 | 108 | 12 | 3 | 932 |
| 36 | Kubo Kimitoshi | 2024 | Eiken H. pylori antigen kit (EIA) | 1 | COI | No | Endoscopy, eradication history, serum antibody, and serum PG II | Ever-infected vs Others | 1055 | 530 | 405 | 4 | 116 |
| 37 | Furuta Takahisa | 2022 | QuickNavi H. pylori (ICA) | - | - | No | Composite: culture, PCR, UBT, RUT, and serum pepsinogen | Current vs Others | 120 | 71 | 0 | 6 | 43 |
| 37 | Furuta Takahisa | 2022 | QuickNavi H. pylori (ICA) | - | - | No | Composite: culture, PCR, UBT, RUT, and serum pepsinogen | Ever-infected vs Others | 120 | 43 | 6 | 0 | 71 |
| 38 | Shin Ito | 2020 | Eiken H. pylori antigen BLEIA | 1 | COI | No | Composite: endoscopy, UBT, and serum IgG antibody | Current vs Others | 133 | 108 | 0 | 2 | 23 |
| 38 | Shin Ito | 2020 | Stool antigen test (EIA; Kit A) | - | - | No | Composite: endoscopy, UBT, and serum IgG antibody | Current vs Others | 133 | 96 | 0 | 14 | 23 |
| 38 | Shin Ito | 2020 | Eiken H. pylori antigen BLEIA | 1 | COI | No | Composite: endoscopy, UBT, and serum IgG antibody | Ever-infected vs Others | 133 | 23 | 2 | 0 | 108 |
| 38 | Shin Ito | 2020 | Stool antigen test (EIA; Kit A) | - | - | No | Composite: endoscopy, UBT, and serum IgG antibody | Ever-infected vs Others | 133 | 23 | 14 | 0 | 96 |
| 39 | Okuda M | 2014 | Testmate Pylori Antigen / TPAg (monoclonal EIA) | 0.1 | COI | No | Quantitative real-time PCR | Current vs Others | 52 | 24 | 0 | 5 | 23 |
| 39 | Okuda M | 2014 | Testmate Pylori Antigen / TPAg (monoclonal EIA) | 0.1 | COI | No | Quantitative real-time PCR | Current vs Others | 99 | 61 | 0 | 5 | 33 |
| 39 | Okuda M | 2014 | Testmate Pylori Antigen / TPAg (monoclonal EIA) | 0.1 | COI | No | Quantitative real-time PCR | Ever-infected vs Others | 52 | 23 | 5 | 0 | 24 |
| 39 | Okuda M | 2014 | Testmate Pylori Antigen / TPAg (monoclonal EIA) | 0.1 | COI | No | Quantitative real-time PCR | Ever-infected vs Others | 99 | 33 | 5 | 0 | 61 |
| 40 | Naomi Sakaedani | 2009 | Premier Platinum HpSA plus (ELISA) | Positive:≥0.120  Negative:<0.10 | OD 450/630 nm | No | SAT (EIA; Testmate) and UBT | Current vs Others | 62 | 29 | 2 | 0 | 31 |
| 40 | Naomi Sakaedani | 2009 | Premier Platinum HpSA plus (ELISA) | Positive:≥0.120  Negative:<0.10 | OD 450/630 nm | No | SAT (EIA; Testmate) and UBT | Ever-infected vs Others | 62 | 31 | 0 | 2 | 29 |
| 41 | Kayoko Asahi | 2007 | Testmate Pylori Antigen EIA (catalase-specific monoclonal) | 0.1 | OD 450/630 nm | No | UBT | Current vs Others | 30 | 12 | 0 | 0 | 18 |
| 41 | Kayoko Asahi | 2007 | Testmate Pylori Antigen EIA (catalase-specific monoclonal) | 0.1 | OD 450/630 nm | No | UBT | Ever-infected vs Others | 30 | 18 | 0 | 0 | 12 |
| 42 | Sakamoto Teruhiko | 2007 | Monoclonal H. pylori stool antigen test (mHpSAT) | 0.1 | OD 450/630 nm | No | Composite: monoclonal SAT, RUT, serum antibody, and histology | Current vs Others | 49 | 39 | 0 | 2 | 8 |
| 42 | Sakamoto Teruhiko | 2007 | Monoclonal H. pylori stool antigen test (mHpSAT) | 0.1 | OD 450/630 nm | No | Composite: monoclonal SAT, RUT, serum antibody, and histology | Ever-infected vs Others | 49 | 8 | 2 | 0 | 39 |
| 43 | Koike Michio | 2005 | Meridian HpSA ELISA | Positive:≥0.120  Negative:<0.10 | OD 450/630 nm | No | UBT | Current vs Others | 43 | 25 | 1 | 0 | 17 |
| 43 | Koike Michio | 2005 | Testmate Pylori Antigen EIA | 0.1 | OD 450/630 nm | No | UBT | Current vs Others | 44 | 25 | 1 | 1 | 17 |
| 43 | Koike Michio | 2005 | Meridian HpSA ELISA | Positive:≥0.120  Negative:<0.10 | OD 450/630 nm | No | UBT | Ever-infected vs Others | 43 | 17 | 0 | 1 | 25 |
| 43 | Koike Michio | 2005 | Testmate Pylori Antigen EIA | 0.1 | OD 450/630 nm | R No | UBT | Ever-infected vs Others | 44 | 17 | 1 | 1 | 25 |

UBT, urea breath test; RUT, rapid urease test; SAT, stool antigen test; HpSA, Helicobacter pylori stool antigen; GI, gastrointestinal; ELISA, enzyme-linked immunosorbent assay; EIA, enzyme immunoassay; LIA, latex immunoassay; CLEIA, chemiluminescent enzyme immunoassay; ICA, immunochromatographic assay; PG, pepsinogen; PCR, polymerase chain reaction; H&E, hematoxylin and eosin

**Supplementary Table 3. Quality assessment of included studies using QUADAS-2**

|  | **Study** | **Risk of Bias** | | | | **Applicability Concerns** | | |
| --- | --- | --- | --- | --- | --- | --- | --- | --- |
| **No.** | **Author (Year)** | **Patient Selection** | **Index Test** | **Reference Standard** | **Flow and Timing** | **Patient Selection** | **Index Test** | **Reference Standard** |
| **Serum antibody** | | | | | | | | |
| 1 | Inoue Kazuhiko (2023) | Low | Low | Low | Low | Low | Low | High |
| 2 | Takayama Takako (2022) | Low | Low | Low | Low | Low | Low | High |
| 3 | Kusano Chika (2021) | Low | Low | Low | Low | Low | Low | High |
| 4 | Ito Masanori (2020) | Low | Low | Low | Low | Low | Unclear | Low |
| 5 | Ueno Yusuke (2020) | Low | Low | Low | Low | Low | Low | High |
| 6 | Kodama Masaaki (2019) | High | Low | Low | Low | High | High | Low |
| 7 | Kawai Sayo (2019) | High | Low | Low | Low | High | Low | High |
| 8 | Tsutsumi Koshiro (2018) | Low | Low | Low | Low | Low | High | High |
| 9 | Miura Mika (2018) | Low | Low | Low | Low | Low | High | High |
| 10 | Kawai Takashi (2018) | Low | Low | Low | Low | Low | Low | Low |
| 11 | Kobayashi Chiaki (2017) | Low | Low | Low | Low | High | Low | Low |
| 12 | Kaihara Kazumi (2017) | High | Low | Low | Low | High | Low | High |
| 13 | Inui Masayuki (2017) | High | Low | Low | Low | High | Low | Low |
| 14 | Aoyama Nobuo (2017) | Low | Low | Low | Low | High | Low | Low |
| 15 | Furuta Takahisa (2016) | Low | Low | High | Low | High | Low | High |
| 16 | Osumi Hiroki (2016) | Low | Low | Low | Low | High | Low | High |
| 17 | Kodama Masaaki (2016) | Low | Low | Low | Low | High | Low | Low |
| 18 | Shiwa Masaaki (2016) | Unclear | Low | High | Low | High | Low | High |
| 19 | Inui Masayuki (2016a) | Low | Low | Low | Low | High | Low | Low |
| 20 | Inui Masayuki (2016b) | Low | Low | Low | Low | High | Low | Low |
| 21 | Inui Masayuki (2016c) | Low | Low | Low | Low | Low | Low | Low |
| 22 | Inoue Kazuhiko (2015) | Low | Low | Low | Low | High | Low | Low |
| 23 | Kodama Masaaki (2015) | High | Low | Low | Low | High | Low | Low |
| 24 | Inui Masayuki (2015) | Low | Low | Low | Low | Low | Low | Low |
| 25 | Furuta Takahisa (2015) | High | Low | High | Low | High | Low | High |
| 26 | Ueda Junko (2014) | Low | Low | Low | Low | Low | Low | High |
| 27 | Iino Chikara (2013) | Low | Low | Low | Low | High | Low | High |
| 28 | Matsuhisa Takeshi (2006) | Low | Low | High | Low | High | Low | High |
| 29 | Okuda Masumi (2005) | High | Low | Low | Low | Low | High | Low |
| **Urine antibody** | | | | | | | | |
| 30 | Mabe Katushiro (2017) | Low | Low | Low | Low | Low | Low | Low |
| 31 | Inoue Kazuhiko (2017) | High | Low | Unclear | Low | High | Low | High |
| 32 | Okuda Masami (2017) | Unclear | Low | High | Low | Low | Low | High |
| 33 | Shimoyama Tadashi (2017) | High | Low | Low | Low | High | Low | Low |
| 34 | Okuda Masumi (2013) | Unclear | Low | Low | Low | Low | Low | Low |
| 35 | Okuda Masumi (2004) | High | Low | Low | Low | Low | Low | Low |
| **Stool antigen** | | | | | | | | |
| 36 | Kubo Kimitoshi (2024) | Low | Low | High | Low | High | Low | High |
| 37 | Furuta Takahisa (2022) | Low | High | Low | Low | High | High | Low |
| 38 | Shin Ito (2020) | Low | Low | Low | Low | High | Low | Low |
| 39 | Okuda M (2014) | High | Low | High | Low | Low | Low | High |
| 40 | Naomi Sakaedani (2009) | Low | Low | Low | Low | High | Low | Low |
| 41 | Kayoko Asahi (2007) | Unclear | Low | Low | Low | High | Low | High |
| 42 | Sakamoto Teruhiko (2007) | Unclear | Low | Low | Low | High | Low | Low |
| 43 | Koike Michio (2005) | Unclear | Low | Low | Low | Low | Low | High |

QUODAS; Quality evaluation of diagnostic accuracy studies

Risk of bias — Patient selection: high if non-consecutive/non-random sampling or case-control design; Index test: high if interpreted with knowledge of the reference standard, or if a post-hoc optimized cutoff was applied; Reference standard: high if only a single reference test was used; Flow and timing: high if not all patients received the same reference standard or if the interval between tests was clinically inappropriate.

Applicability concerns — Patient selection: high if previously infected (eradicated) patients were included; Index test: high if a research-grade assay not widely available in clinical practice was used; Reference standard: high if only a single reference test was used.

**Supplementary Table 4. Subgroup analysis by presence of previously infected individuals**

| **Subgroup** | **Index test** | **Diagnostic objective** | **Sn (95% CI)** | **Sp (95% CI)** | **LR+ (95% CI)** | **LR- (95% CI)** | **DOR (95% CI)** |
| --- | --- | --- | --- | --- | --- | --- | --- |
| With previously infected individuals | Serum antibody | Current vs Others | 0.876 (0.712-0.953) | 0.861 (0.705-0.942) | 6.318 (2.567-15.552) | 0.144 (0.053-0.391) | 43.908 (7.471-258.044) |
| Without previously infected individuals | Serum antibody | Current vs Others | 0.929 (0.901-0.950) | 0.967 (0.934-0.984) | 28.231 (13.977-57.021) | 0.074 (0.053-0.103) | 383.562 (181.098-812.377) |
| With previously infected individuals | Serum antibody | Ever-infected vs Others | 0.858 (0.632-0.955) | 0.938 (0.813-0.982) | 13.929 (4.894-39.643) | 0.152 (0.055-0.420) | 91.937 (39.400-214.526) |
| Without previously infected individuals | Serum antibody | Ever-infected vs Others | 0.929 (0.901-0.950) | 0.967 (0.934-0.984) | 28.231 (13.977-57.021) | 0.074 (0.053-0.103) | 383.562 (181.098-812.377) |
| Without previously infected individuals | Urine antibody | Current vs Others | 0.885 (0.710-0.960) | 0.970 (0.728-0.997) | 29.016 (2.755-305.556) | 0.119 (0.045-0.319) | 243.618 (24.319-2440.490) |
| Without previously infected individuals | Urine antibody | Ever-infected vs Others | 0.885 (0.710-0.960) | 0.970 (0.728-0.997) | 29.016 (2.755-305.556) | 0.119 (0.045-0.319) | 243.618 (24.319-2440.490) |
| ^a^With previously infected individuals | Stool antigen | Current vs Others | 0.973 (0.923-0.994) | 0.987 (0.978-0.993) | 76.541 (43.588-134.405) | 0.027 (0.009-0.084) | 2796.000 (776.816-10063.664) |
| Without previously infected individuals | Stool antigen | Current vs Others | 0.933 (0.899-0.956) | 0.983 (0.923-0.997) | 55.998 (11.799-265.761) | 0.068 (0.045-0.101) | 826.004 (203.708-3349.324) |
| ^a^With previously infected individuals | Stool antigen | Ever-infected vs Others | 0.223 (0.188-0.261) | 0.993 (0.981-0.998) | 29.724 (11.051-79.945) | 0.783 (0.748-0.821) | 37.951 (13.890-103.687) |
| Without previously infected individuals | Stool antigen | Ever-infected vs Others | 0.933 (0.899-0.956) | 0.983 (0.923-0.997) | 55.998 (11.799-265.761) | 0.068 (0.045-0.101) | 826.004 (203.708-3349.324) |

Sn, sensitivity; Sp, specificity; LR+, Positive likelihood ratio; LR-, Negative likelihood ratio; DOR, Diagnostic odds ratio; CI, confidence interval

^a^Single study only (not pooled); values represent individual study estimates.

In studies without previously infected individuals, Current vs Others and Ever-infected vs Others yield identical results for all index tests because current infection and ever-infected status define the same classification.

**Supplementary Table 5. Subgroup analysis: post-hoc cutoff use**

| **Subgroup** | **Index test** | **Diagnostic objective** | **Sn (95% CI)** | **Sp (95% CI)** | **LR+ (95% CI)** | **LR- (95% CI)** | **DOR (95% CI)** |
| --- | --- | --- | --- | --- | --- | --- | --- |
| No | Serum antibody | Current vs Others | 0.926 (0.902-0.944) | 0.961 (0.931-0.978) | 23.825 (13.329-42.586) | 0.077 (0.059-0.102) | 308.277 (163.040-582.893) |
| Yes | Serum antibody | Current vs Others | 0.886 (0.552-0.980) | 0.836 (0.601-0.946) | 5.416 (1.647-17.810) | 0.136 (0.023-0.815) | 39.690 (2.219-709.844) |
| No | Serum antibody | Ever-infected vs Others | 0.908 (0.869-0.937) | 0.963 (0.935-0.979) | 24.349 (13.943-42.519) | 0.095 (0.066-0.137) | 255.955 (136.942-478.397) |
| Yes | Serum antibody | Ever-infected vs Others | 0.899 (0.813-0.948) | 0.905 (0.749-0.968) | 9.444 (3.437-25.953) | 0.112 (0.062-0.202) | 84.352 (31.966-222.590) |
| No | Urine antibody | Current vs Others | 0.885 (0.710-0.960) | 0.970 (0.728-0.997) | 29.016 (2.755-305.556) | 0.119 (0.045-0.319) | 243.618 (24.319-2440.490) |
| No | Urine antibody | Ever-infected vs Others | 0.885 (0.710-0.960) | 0.970 (0.728-0.997) | 29.016 (2.755-305.556) | 0.119 (0.045-0.319) | 243.618 (24.319-2440.490) |
| No | Stool antigen | Current vs Others | 0.928 (0.883-0.956) | 0.972 (0.937-0.988) | 33.044 (14.427-75.685) | 0.074 (0.045-0.122) | 443.767 (161.998-1215.627) |
| No | Stool antigen | Ever-infected vs Others | 0.934 (0.736-0.986) | 0.992 (0.948-0.999) | 119.887 (18.184-790.433) | 0.066 (0.014-0.301) | 1815.289 (271.880-12120.308) |

Sn, sensitivity; Sp, specificity; LR+, Positive likelihood ratio; LR-, Negative likelihood ratio; DOR, Diagnostic odds ratio; CI, confidence interval

Post-hoc optimal cutoff subgroup analysis was not performed for urine antibody and stool antigen due to insufficient studies with post-hoc cutoff values.

**Supplementary Table 6a. Subgroup analysis: test kit type**

| **Subgroup** | **Index test** | **Diagnostic objective** | **Sn** **(95% CI)** | **Sp (95% CI)** | **LR+ (95% CI)** | **LR- (95% CI)** | **DOR (95% CI)** |
| --- | --- | --- | --- | --- | --- | --- | --- |
| Serum EIA  E-plate | Serum antibody | Current vs Others | 0.915 (0.884-0.938) | 0.993 (0.962-0.999) | 134.498 (23.648-764.966) | 0.086 (0.063-0.117) | 1562.434 (284.360-8584.899) |
| Serum EIA  non-E-plate | Serum antibody | Current vs Others | 0.959 (0.911-0.981) | 0.933 (0.749-0.985) | 14.413 (3.367-61.701) | 0.044 (0.020-0.100) | 324.927 (48.884-2159.735) |
| Serum PA/LA | Serum antibody | Current vs Others | 0.923 (0.878-0.952) | 0.937 (0.897-0.962) | 14.545 (8.756-24.160) | 0.082 (0.051-0.134) | 176.514 (75.569-412.299) |
| Serum EIA  E-plate | Serum antibody | Ever-infected vs Others | 0.925 (0.885-0.952) | 0.994 (0.958-0.999) | 149.733 (21.679-1034.189) | 0.076 (0.049-0.116) | 1982.076 (352.779-11136.236) |
| Serum EIA  non-E-plate | Serum antibody | Ever-infected vs Others | 0.944 (0.850-0.981) | 0.952 (0.790-0.990) | 19.506 (3.992-95.317) | 0.059 (0.021-0.168) | 331.756 (39.691-2772.948) |
| Serum PA/LA | Serum antibody | Ever-infected vs Others | 0.906 (0.839-0.947) | 0.944 (0.902-0.969) | 16.162 (9.258-28.214) | 0.099 (0.057-0.173) | 162.861 (78.020-339.964) |
| Urine EIA | Urine antibody | Current vs Others | 0.940 (0.880-0.971) | 0.947 (0.890-0.975) | 17.763 (8.174-38.604) | 0.063 (0.030-0.133) | 281.378 (70.679-1120.181) |
| Urine ICA | Urine antibody | Current vs Others | 0.794 (0.465-0.945) | 0.974 (0.510-0.999) | 30.682 (1.033-911.439) | 0.212 (0.067-0.669) | 144.778 (5.237-4002.689) |
| Urine EIA | Urine antibody | Ever-infected vs Others | 0.940 (0.880-0.971) | 0.947 (0.890-0.975) | 17.763 (8.174-38.604) | 0.063 (0.030-0.133) | 281.378 (70.679-1120.181) |
| Urine ICA | Urine antibody | Ever-infected vs Others | 0.794 (0.465-0.945) | 0.974 (0.510-0.999) | 30.682 (1.033-911.439) | 0.212 (0.067-0.669) | 144.778 (5.237-4002.689) |
| Stool EIA | Stool antigen | Current vs Others | 0.954 (0.910-0.977) | 0.987 (0.971-0.994) | 72.450 (32.849-159.793) | 0.047 (0.023-0.092) | 1555.419 (681.447-3550.281) |
| ^a^Stool ICA | Stool antigen | Current vs Others | 0.922 (0.838-0.971) | 1.000 (0.918-1.000) | N/A | 0.078 (0.029-0.176) | N/A |
| Stool EIA | Stool antigen | Ever-infected vs Others | 0.938 (0.681-0.991) | 0.991 (0.931-0.999) | 102.601 (13.706-768.057) | 0.063 (0.010-0.390) | 1626.686 (178.451-14828.174) |
| ^a^Stool ICA | Stool antigen | Ever-infected vs Others | 0.922 (0.838-0.971) | 1.000 (0.918-1.000) | N/A | 0.078 (0.029-0.176) | N/A |

Sn, sensitivity; Sp, specificity; LR+, Positive likelihood ratio; LR-, Negative likelihood ratio; DOR, Diagnostic odds ratio; CI, confidence interval; EIA = Enzyme immunoassay; ICA = Immunochromatographic assay; PA/LA = Particle agglutination/Latex agglutination; N/A = Not available

^a^Single study only (not pooled); values represent individual study estimates.

**Supplementary Table 6b. Subgroup analysis of serum antibody tests by test kit type after stratification by the presence of previously infected individuals**

| **Test kit type** | **Previously infected**  **individuals** | **Diagnostic objective** | **Sn (95% CI)** | **Sp (95% CI)** | **LR+ (95% CI)** | **LR− (95% CI)** | **DOR (95% CI)** |
| --- | --- | --- | --- | --- | --- | --- | --- |
| Serum EIA E-plate | With | Current vs Others | 0.871 (0.813-0.913) | 0.962 (0.910-0.984) | 22.814 (9.846-52.858) | 0.134 (0.094-0.192) | 170.153 (86.320-335.405) |
| Serum EIA E-plate | Without | Current vs Others | 0.918 (0.886-0.941) | 0.997 (0.965-1.000) | 265.712 (26.179-2696.905) | 0.082 (0.059-0.114) | 3226.006 (349.140-29807.851) |
| Serum EIA non-E-plate | With | Current vs Others | 0.941 (0.936-0.945) | 0.866 (0.840-0.888) | 6.995 (5.858-8.353) | 0.069 (0.063-0.074) | 102.105 (83.411-124.988) |
| Serum EIA non-E-plate | Without | Current vs Others | 0.966 (0.893-0.990) | 0.969 (0.717-0.997) | 31.593 (2.719-367.042) | 0.035 (0.011-0.115) | 895.158 (48.686-16458.743) |
| Serum PA/LA | With | Current vs Others | 0.860 (0.620-0.959) | 0.847 (0.633-0.947) | 5.632 (1.869-16.972) | 0.165 (0.047-0.576) | 34.080 (3.787-306.700) |
| Serum PA/LA | Without | Current vs Others | 0.935 (0.884-0.965) | 0.950 (0.914-0.971) | 18.533 (10.713-32.061) | 0.068 (0.037-0.125) | 272.626 (110.367-673.431) |

Sn, sensitivity; Sp, specificity; LR+, Positive likelihood ratio; LR-, Negative likelihood ratio; DOR, Diagnostic odds ratio; CI, confidence interval; EIA = Enzyme immunoassay; ICA = Immunochromatographic assay; PA/LA = Particle agglutination/Latex agglutination.

**Supplementary Table 6c. Additional analysis of stool antigen EIA tests after separating BLEIA and excluding polyclonal HpSA**

| **Subgroup** | **Diagnostic objective** | **Sn (95% CI)** | **Sp (95% CI)** | **LR+ (95% CI)** | **LR− (95% CI)** | **DOR (95% CI)** |
| --- | --- | --- | --- | --- | --- | --- |
| BLEIA | Current vs Others | 0.977 (0.961-0.987) | 0.988 (0.984-0.990) | 78.760 (62.356-99.478) | 0.023 (0.013-0.040) | 3437.845 (1705.572-6929.508) |
| EIA excluding BLEIA | Current vs Others | 0.920 (0.847-0.960) | 0.977 (0.899-0.995) | 40.025 (8.929-179.415) | 0.082 (0.043-0.157) | 489.557 (128.396-1866.619) |
| EIA excluding BLEIA and polyclonal HpSA | Current vs Others | 0.914 (0.850-0.952) | 0.981 (0.898-0.997) | 47.513 (8.656-260.802) | 0.088 (0.050-0.154) | 540.233 (112.704-2589.541) |
| BLEIA | Ever-infected vs Others | 0.797 (0.003-1.000) | 0.993 (0.984-0.997) | 108.746 (19.573-604.186) | 0.204 (0.001-55.410) | 532.509 (0.408-695272.273) |
| EIA excluding BLEIA | Ever-infected vs Others | 0.920 (0.847-0.960) | 0.977 (0.899-0.995) | 40.025 (8.929-179.415) | 0.082 (0.043-0.157) | 489.557 (128.396-1866.619) |
| EIA excluding BLEIA and polyclonal HpSA | Ever-infected vs Others | 0.914 (0.850-0.952) | 0.981 (0.898-0.997) | 47.513 (8.656-260.802) | 0.088 (0.050-0.154) | 540.233 (112.704-2589.541) |

Sn, sensitivity; Sp, specificity; LR+, positive likelihood ratio; LR−, negative likelihood ratio; DOR, diagnostic odds ratio; CI, confidence interval; BLEIA, bioluminescent enzyme immunoassay; EIA, enzyme immunoassay; HpSA, Helicobacter pylori stool antigen. Polyclonal HpSA refers to a polyclonal antibody-based stool antigen EIA.

**Supplementary Table 7. Subgroup analysis: age group**

| **Subgroup** | **Index test** | **Diagnostic objective** | **Sn (95% CI)** | **Sp (95% CI)** | **LR+ (95% CI)** | **LR- (95% CI)** | **DOR (95% CI)** |
| --- | --- | --- | --- | --- | --- | --- | --- |
| Adults | Serum antibody | Current vs Others | 0.928 (0.903-0.947) | 0.949 (0.912-0.971) | 18.242 (10.441-31.872) | 0.076 (0.056-0.104) | 240.303 (116.907-493.943) |
| Children | Serum antibody | Current vs Others | 0.827 (0.515-0.956) | 0.980 (0.466-1.000) | 42.146 (0.876-2027.590) | 0.176 (0.052-0.594) | 239.152 (5.813-9839.043) |
| Adults | Serum antibody | Ever-infected vs Others | 0.914 (0.875-0.942) | 0.957 (0.926-0.975) | 21.049 (12.304-36.009) | 0.090 (0.062-0.132) | 233.574 (123.246-442.668) |
| Children | Serum antibody | Ever-infected vs Others | 0.827 (0.515-0.956) | 0.980 (0.466-1.000) | 42.146 (0.876-2027.590) | 0.176 (0.052-0.594) | 239.152 (5.813-9839.043) |
| Adults | Urine antibody | Current vs Others | 0.895 (0.766-0.957) | 0.872 (0.469-0.981) | 7.016 (1.111-44.300) | 0.121 (0.043-0.335) | 58.195 (3.917-864.500) |
| Children | Urine antibody | Current vs Others | 0.894 (0.747-0.960) | 0.973 (0.943-0.987) | 32.920 (16.389-66.128) | 0.109 (0.043-0.275) | 301.419 (128.592-706.520) |
| Adults | Urine antibody | Ever-infected vs Others | 0.895 (0.766-0.957) | 0.872 (0.469-0.981) | 7.016 (1.111-44.300) | 0.121 (0.043-0.335) | 58.195 (3.917-864.500) |
| Children | Urine antibody | Ever-infected vs Others | 0.894 (0.747-0.960) | 0.973 (0.943-0.987) | 32.920 (16.389-66.128) | 0.109 (0.043-0.275) | 301.419 (128.592-706.520) |
| Adults | Stool antigen | Current vs Others | 0.938 (0.900-0.963) | 0.989 (0.979-0.994) | 84.759 (46.107-155.816) | 0.062 (0.038-0.102) | 1357.907 (712.207-2589.011) |
| Children | Stool antigen | Current vs Others | 0.933 (0.494-0.995) | 0.980 (0.770-0.999) | 45.833 (3.926-535.082) | 0.068 (0.006-0.780) | 673.778 (175.326-2589.329) |
| Adults | Stool antigen | Ever-infected vs Others | 0.823 (0.385-0.972) | 0.984 (0.944-0.995) | 50.095 (15.310-163.916) | 0.180 (0.035-0.931) | 278.579 (38.047-2039.752) |
| Children | Stool antigen | Ever-infected vs Others | 0.919 (0.490-0.993) | 0.965 (0.812-0.994) | 26.417 (5.307-131.486) | 0.084 (0.009-0.763) | 316.366 (125.146-799.764) |

Sn, sensitivity; Sp, specificity; LR+, Positive likelihood ratio; LR-, Negative likelihood ratio; DOR, Diagnostic odds ratio; CI, confidence interval

**Supplementary Table 8. Subgroup analysis: reference standard composition**

| **Subgroup** | **Index test** | **Diagnostic objective** | **Sn (95% CI)** | **Sp (95% CI)** | **LR+ (95% CI)** | **LR- (95% CI)** | **DOR (95% CI)** |
| --- | --- | --- | --- | --- | --- | --- | --- |
| Multiple standards | Serum antibody | Current vs Others | 0.916 (0.888-0.938) | 0.943 (0.886-0.972) | 16.009 (7.891-32.481) | 0.089 (0.066-0.120) | 179.828 (80.059-403.925) |
| Single standard | Serum antibody | Current vs Others | 0.923 (0.868-0.956) | 0.965 (0.899-0.988) | 26.052 (8.863-76.582) | 0.080 (0.046-0.139) | 326.948 (95.303-1121.630) |
| Multiple standards | Serum antibody | Ever-infected vs Others | 0.906 (0.840-0.947) | 0.948 (0.894-0.975) | 17.330 (8.546-35.140) | 0.099 (0.057-0.170) | 175.563 (79.469-387.856) |
| Single standard | Serum antibody | Ever-infected vs Others | 0.909 (0.845-0.949) | 0.971 (0.924-0.990) | 31.849 (11.790-86.039) | 0.093 (0.054-0.162) | 341.518 (117.196-995.211) |
| Multiple standards | Urine antibody | Current vs Others | 0.920 (0.770-0.975) | 0.946 (0.761-0.990) | 17.052 (3.303-88.035) | 0.084 (0.026-0.271) | 202.067 (19.082-2139.794) |
| Single standard | Urine antibody | Current vs Others | 0.806 (0.304-0.975) | 0.978 (0.781-0.998) | 36.817 (4.697-288.601) | 0.198 (0.034-1.157) | 186.066 (85.457-405.120) |
| Multiple standards | Urine antibody | Ever-infected vs Others | 0.920 (0.770-0.975) | 0.946 (0.761-0.990) | 17.052 (3.303-88.035) | 0.084 (0.026-0.271) | 202.067 (19.082-2139.794) |
| Single standard | Urine antibody | Ever-infected vs Others | 0.806 (0.304-0.975) | 0.978 (0.781-0.998) | 36.817 (4.697-288.601) | 0.198 (0.034-1.157) | 186.084 (85.445-405.261) |
| Multiple standards | Stool antigen | Current vs Others | 0.935 (0.893-0.961) | 0.985 (0.817-0.999) | 60.750 (4.489-822.124) | 0.066 (0.041-0.109) | 914.704 (75.992-11010.156) |
| Single standard | Stool antigen | Current vs Others | 0.952 (0.864-0.984) | 0.976 (0.927-0.992) | 39.155 (12.861-119.211) | 0.050 (0.017-0.145) | 788.474 (174.963-3553.285) |
| Multiple standards | Stool antigen | Ever-infected vs Others | 0.935 (0.893-0.961) | 0.985 (0.817-0.999) | 60.750 (4.489-822.124) | 0.066 (0.041-0.109) | 914.704 (75.992-11010.156) |
| Single standard | Stool antigen | Ever-infected vs Others | 0.895 (0.233-0.996) | 0.993 (0.840-1.000) | 123.610 (4.966-3077.069) | 0.106 (0.005-2.083) | 1165.727 (14.792-91865.675) |

Sn, sensitivity; Sp, specificity; LR+, Positive likelihood ratio; LR-, Negative likelihood ratio; DOR, Diagnostic odds ratio; CI, confidence interval.

**Supplementary Table 9. Analysis under clinically relevant conditions**

| **Index test** | **Diagnostic objective** | **Sn (95% CI)** | **Sp (95% CI)** | **LR+ (95% CI)** | **LR- (95% CI)** | **DOR (95% CI)** |
| --- | --- | --- | --- | --- | --- | --- |
| Serum | Current vs Others | 0.908 (0.855-0.943) | 0.968 (0.928-0.986) | 28.313 (12.211-65.651) | 0.095 (0.059-0.153) | 298.599 (97.344-915.942) |
| Urine | Current vs Others | 0.920 (0.770-0.975) | 0.946 (0.761-0.990) | 17.052 (3.303-88.035) | 0.084 (0.026-0.271) | 202.067 (19.082-2139.794) |
| Stool | Current vs Others | 0.935 (0.893-0.961) | 0.985 (0.817-0.999) | 60.750 (4.489-822.124) | 0.066 (0.041-0.109) | 914.704 (75.992-11010.156) |

Sn, sensitivity; Sp, specificity; LR+, Positive likelihood ratio; LR-, Negative likelihood ratio; DOR, Diagnostic odds ratio; CI, confidence interval

Because previously infected individuals were not present, results for ever-infected versus others were identical and are not shown.

**Supplementary Figure 1. Summary ROC curves of serum antibody tests**

(a) Current infection versus others; (b) ever-infected versus others. Each circle represents a study-level estimate of sensitivity and specificity; circle size is proportional to study sample size. Sn, sensitivity; Sp, specificity; SROC, summary receiver operating characteristic; AUC, area under the curve.

**Supplementary Figure 2. Summary ROC curve of urine antibody tests**

As previously infected patients were not included, results for current infection versus others and ever-infected versus others are identical; only current infection versus others is presented. Each circle represents a study-level estimate of sensitivity and specificity; circle size is proportional to study sample size. Sn, sensitivity; Sp, specificity; SROC, summary receiver operating characteristic; AUC, area under the curve.

**Supplementary Figure 3. Summary ROC curves of stool antigen tests**

(a) Current infection versus others; (b) ever-infected versus others. Each circle represents a study-level estimate of sensitivity and specificity; circle size is proportional to study sample size. Sn, sensitivity; Sp, specificity; SROC, summary receiver operating characteristic; AUC, area under the curve.

**Supplementary Figure 4. Forest plots: serum antibody, studies with previously infected individuals**

a. current infection versus others; b. ever-infected versus others. τ²sn and τ²sp represents variances of random effects for sensitivity and specificity. TP, true positive; FP, false positive; FN, false negative; TN, true negative; CI, confidence interval; GLM, generalized linear model (fixed effects only), LMM-UN, linear mixed-effects model incorporating random effects with an unstructured variance-covariance matrix.

Supplementary Figure 5. Forest plots: serum antibody, studies without previously infected individuals

τ²sn and τ²sp represents variances of random effects for sensitivity and specificity. TP, true positive; FP, false positive; FN, false negative; TN, true negative; CI, confidence interval; GLMM-UN, generalized linear mixed-effects model incorporating random effects with an unstructured variance-covariance matrix.

Because previously infected individuals were not present, the diagnostic classifications for current infection versus others and ever-infected status were identical; therefore, only current infection versus others is presented.

**Supplementary Figure 6. Forest plots: serum antibody, without post-hoc cutoff use**

a. current infection versus others; b. ever-infected versus others. τ²sn and τ²sp represents variances of random effects for sensitivity and specificity. TP, true positive; FP, false positive; FN, false negative; TN, true negative; CI, confidence interval; GLMM-UN, generalized linear mixed-effects model incorporating random effects with an unstructured variance-covariance matrix.

**Supplementary Figure 7. Forest plots: serum antibody, with post-hoc cutoff use**

a. current infection versus others; b. ever-infected versus others. τ²sn and τ²sp represents variances of random effects for sensitivity and specificity. TP, true positive; FP, false positive; FN, false negative; TN, true negative; CI, confidence interval; GLMM-EQ, generalized linear mixed-effects model with random effects assuming equal variance for sensitivity and specificity; LMM-EQ, linear mixed-effects model with random effects assuming equal variance for sensitivity and specificity.

**Supplementary Figure 8. Forest plots: serum antibody, EIA (E-plate)**

a. current infection versus others; b. ever-infected versus others. τ²sn and τ²sp represents variances of random effects for sensitivity and specificity. TP, true positive; FP, false positive; FN, false negative; TN, true negative; EIA, enzyme immunoassay; CI, confidence interval; GLMM-UN, generalized linear mixed-effects model incorporating random effects with an unstructured variance-covariance matrix

**Supplementary Figure 9. Forest plots: serum antibody, EIA (non-E-plate)**

a. current infection versus others; b. ever-infected versus others. τ²sn and τ²sp represents variances of random effects for sensitivity and specificity. TP, true positive; FP, false positive; FN, false negative; TN, true negative; EIA, enzyme immunoassay; CI, confidence interval; GLMM-UN, generalized linear mixed-effects model incorporating random effects with an unstructured variance-covariance matrix

**Supplementary Figure 10. Forest plots: serum antibody, PA/LA**

a. current infection versus others; b. ever-infected versus others. τ²sn and τ²sp represents variances of random effects for sensitivity and specificity. TP, true positive; FP, false positive; FN, false negative; TN, true negative; PA/LA, particle agglutination/latex agglutination; CI, confidence interval; GLMM-UN, generalized linear mixed-effects model incorporating random effects with an unstructured variance-covariance matrix

**Supplementary Figure 11. Forest plots: urine antibody, EIA**

τ²sn and τ²sp represents variances of random effects for sensitivity and specificity. TP, true positive; FP, false positive; FN, false negative; TN, true negative; EIA, enzyme immunoassay; CI, confidence interval; LMM-EQ, linear mixed-effects model with random effects assuming equal variance for sensitivity and specificity

Because previously infected individuals were not present, the diagnostic classifications for current infection versus others and ever-infected status were identical; therefore, only current infection versus others is presented.

**Supplementary Figure 12. Forest plots: urine antibody, ICA**

τ²sn and τ²sp represents variances of random effects for sensitivity and specificity. TP, true positive; FP, false positive; FN, false negative; TN, true negative; ICA, immunochromatographic assay; CI, confidence interval; GLMM-EQ, generalized linear mixed-effects model with random effects assuming equal variance for sensitivity and specificity

Because previously infected individuals were not present, the diagnostic classifications for current infection versus others and ever-infected status were identical; therefore, only current infection versus others is presented.

**Supplementary Figure 13. Forest plots: stool antigen, EIA**

a. current infection versus others; b. ever-infected versus others. τ²sn and τ²sp represents variances of random effects for sensitivity and specificity. TP, true positive; FP, false positive; FN, false negative; TN, true negative; EIA, enzyme immunoassay; CI, confidence interval; GLMM-EQ, generalized linear mixed-effects model with random effects assuming equal variance for sensitivity and specificity

**Supplementary Figure 14. Forest plots: serum antibody, adults**

a. current infection versus others; b. ever-infected versus others. τ²sn and τ²sp represents variances of random effects for sensitivity and specificity. TP, true positive; FP, false positive; FN, false negative; TN, true negative; CI, confidence interval; GLMM-UN, generalized linear mixed-effects model incorporating random effects with an unstructured variance-covariance matrix

**Supplementary Figure 15. Forest plots: serum antibody, children**

a. current infection versus others; b. ever-infected versus others. τ²sn and τ²sp represents variances of random effects for sensitivity and specificity. TP, true positive; FP, false positive; FN, false negative; TN, true negative; CI, confidence interval; GLMM-UN, generalized linear mixed-effects model incorporating random effects with an unstructured variance-covariance matrix

**Supplementary Figure 16. Forest plots: urine antibody, adults**

a. current infection versus others; b. ever-infected versus others. τ²sn and τ²sp represents variances of random effects for sensitivity and specificity. TP, true positive; FP, false positive; FN, false negative; TN, true negative; CI, confidence interval; GLMM-EQ, generalized linear mixed-effects model with random effects assuming equal variance for sensitivity and specificity

**Supplementary Figure 17. Forest plots: urine antibody, children**

a. current infection versus others; b. ever-infected versus others. TP, true positive; FP, false positive; FN, false negative; TN, true negative; CI, confidence interval; GLM, generalized linear model (fixed effects only)

**Supplementary Figure 18. Forest plots: stool antigen, adults**

a. current infection versus others; b. ever-infected versus others. τ²sn and τ²sp represents variances of random effects for sensitivity and specificity. TP, true positive; FP, false positive; FN, false negative; TN, true negative; CI, confidence interval; GLM, generalized linear model (fixed effects only); LMM-EQ, linear mixed-effects model with random effects assuming equal variance for sensitivity and specificity

**Supplementary Figure 19. Forest plots: stool antigen, children**

a. current infection versus others; b. ever-infected versus others. τ²sn and τ²sp represents variances of random effects for sensitivity and specificity. TP, true positive; FP, false positive; FN, false negative; TN, true negative; CI, confidence interval; GLM-EQ, generalized linear model assuming equal variance for sensitivity and specificity, LMM-EQ, linear mixed-effects model with random effects assuming equal variance for sensitivity and specificity.

**Supplementary Figure 20. Forest plots: serum antibody, composite reference standards**

a. current infection versus others; b. ever-infected versus others. τ²sn and τ²sp represents variances of random effects for sensitivity and specificity. TP, true positive; FP, false positive; FN, false negative; TN, true negative; CI, confidence interval; GLMM-UN, generalized linear mixed-effects model incorporating random effects with an unstructured variance-covariance matrix

**Supplementary Figure 21. Forest plots: serum antibody, single reference standard**

a. current infection versus others; b. ever-infected versus others. τ²sn and τ²sp represents variances of random effects for sensitivity and specificity. TP, true positive; FP, false positive; FN, false negative; TN, true negative; CI, confidence interval; GLMM-UN, generalized linear mixed-effects model incorporating random effects with an unstructured variance-covariance matrix

**Supplementary Figure 22. Forest plots: urine antibody, composite reference standards**

a. current infection versus others; b. ever-infected versus others. TP, true positive; FP, false positive; FN, false negative; TN, true negative; CI, confidence interval; GLM-EQ, generalized linear model assuming equal variance for sensitivity and specificity.

**Supplementary Figure 23. Forest plots: urine antibody, single reference standard**

a. current infection versus others; b. ever-infected versus others. τ²sn and τ²sp represents variances of random effects for sensitivity and specificity. TP, true positive; FP, false positive; FN, false negative; TN, true negative; CI, confidence interval; GLMM-EQ, generalized linear mixed-effects model with random effects assuming equal variance for sensitivity and specificity

**Supplementary Figure 24. Forest plots: stool antigen, composite reference standards**

a. current infection versus others; b. ever-infected versus others. TP, true positive; FP, false positive; FN, false negative; TN, true negative; CI, confidence interval; GLM, generalized linear model (fixed effects only).

**Supplementary Figure 25. Forest plots: stool antigen, single reference standard**

a.	Current infection versus others

b.	Ever-infected versus others

τ²sn and τ²sp represents variances of random effects for sensitivity and specificity. TP, true positive; FP, false positive; FN, false negative; TN, true negative; CI, confidence interval; GLMM-UN, generalized linear mixed-effects model incorporating random effects with an unstructured variance-covariance matrix. ; GLMM-EQ, generalized linear mixed-effects model with random effects assuming equal variance for sensitivity and specificity.

**Supplementary Figure 26. Forest plots: serum antibody, clinically relevant conditions**

a. current infection versus others; b. ever-infected versus others. τ²sn and τ²sp represents variances of random effects for sensitivity and specificity. TP, true positive; FP, false positive; FN, false negative; TN, true negative; CI, confidence interval; GLMM-UN, generalized linear mixed-effects model incorporating random effects with an unstructured variance-covariance matrix.

**Supplementary Figure 27. SROC curve: serum antibody, clinically relevant conditions**

a. current infection versus others; b. ever-infected versus others. ROC, receiver operating characteristic; Sn, sensitivity; Sp, specificity; SROC, Summary Receiver Operating Characteristic

Summary ROC curves were generated using the bivariate random-effects model. Each circle represents a study-level estimate; circle size is proportional to sample size. The summary operating point (diamond) and 95% confidence region are shown.

**Supplementary Figure 28. Forest plots: urine antibody, clinically relevant conditions**

a. current infection versus others; b. ever-infected versus others. τ²sn and τ²sp represents variances of random effects for sensitivity and specificity. TP, true positive; FP, false positive; FN, false negative; TN, true negative; CI, confidence interval; GLMM-EQ, generalized linear mixed-effects model with random effects assuming equal variance for sensitivity and specificity.

**Supplementary Figure 29. SROC curve: urine antibody, clinically relevant conditions**

a. current infection versus others; b. ever-infected versus others. ROC, receiver operating characteristic; Sn, sensitivity; Sp, specificity; SROC, summary receiver operating characteristic

Summary ROC curves were generated using the bivariate random-effects model. Each circle represents a study-level estimate; circle size is proportional to sample size. The summary operating point (diamond) and 95% confidence region are shown.

**Supplementary Figure 30. Forest plots: stool antigen, clinically relevant conditions**

a. current infection versus others; b. ever-infected versus others. TP, true positive; FP, false positive; FN, false negative; TN, true negative; CI, confidence interval; GLM, generalized linear model (fixed effects only).
